# Fixed point results in intuitionistic fuzzy pentagonal controlled metric spaces with applications to dynamic market equilibrium and satellite web coupling

**DOI:** 10.1371/journal.pone.0303141

**Published:** 2024-08-28

**Authors:** Umar Ishtiaq, Salha Alshaikey, Muhammad Bilal Riaz, Khaleel Ahmad

**Affiliations:** 1 Office of Research, Innovation and Commercialization, University of Management and Technology, Lahore, Pakistan; 2 Mathematics Department, Al-Qunfudah University College, Umm Al-Qura University, Mecca, Saudi Arabia; 3 IT4Innovations, VSB – Technical University of Ostrava, Ostrava, Czech Republic; 4 Department of Computer Science and Mathematics, Lebanese American University, Byblos, Lebanon; 5 Department of Mathematics, University of Management and Technology, Lahore, Pakistan; Korea National University of Transportation, REPUBLIC OF KOREA

## Abstract

This manuscript contains several new spaces as the generalizations of fuzzy triple controlled metric space, fuzzy controlled hexagonal metric space, fuzzy pentagonal controlled metric space and intuitionistic fuzzy double controlled metric space. We prove the Banach fixed point theorem in the context of intuitionistic fuzzy pentagonal controlled metric space, which generalizes the previous ones in the existing literature. Further, we provide several non-trivial examples to support the main results. The capacity of intuitionistic fuzzy pentagonal controlled metric spaces to model hesitation, capture dual information, handle imperfect information, and provide a more nuanced representation of uncertainty makes them important in dynamic market equilibrium. In the context of changing market dynamics, these aspects contribute to a more realistic and flexible modelling approach. We present an application to dynamic market equilibrium and solve a boundary value problem for a satellite web coupling.

## 1. Introduction

Fuzzy sets (FSs) are very beneficial when dealing with data or information that contains uncertainty or ambiguity. They give a framework for dealing with circumstances in which the borders between categories are hazy or ambiguous. Fuzzy sets are therefore useful in fields such as artificial intelligence, expert systems, decision-making, and control systems. Zadeh [1] presented FSs as an extension of classical set theory in 1965. Unlike classical sets, which are binary and feature elements that either belong or do not belong to the set, FSs allow for degrees of membership. In other words, an element can have partial membership in a FS, which is represented by a value between 0 and 1, indicating the degree to which it belongs to the set.

In 1979, Itoh [2] proved fixed point theorems with an application to random differential equations in Banach spaces. Schweizer and Saklar [3] itroduced the notion of continuous t-norms (CTNs). Kramosil and Michálek [4] introduced the concept of fuzzy metric space (FMS) by utilizing CTNs. George and Veeramani [5] modify the notion of FMS and presented Hausdorff topology in FMS. Grabiec [6] proved the Banach contraction theorem and Edelstein theorem in FMS. Han [7] demonstrated Banach fixed point theorem from the point of view of digital topology. Kamran et al. [8] developed the extended b-metric space and demonstrated numerous fixed point findings for contraction mappings. Mehmood et al. [9] proposed and demonstrated fixed point theorems for fuzzy rectangular b-metric spaces. Badshah-e-Rome et al. [10] defined extended fuzzy rectangular b-metric spaces and demonstrated numerous fixed point findings using *α*-admissibility. Furqan et al. [11] defined fuzzy triple controlled metric spaces (FTCMSs) as a generalization of various spaces. Zubair et al. [12] introduced and proved the Banach fixed point result for fuzzy extended hexagonal b-metric spaces (FEHbMSs). Hussain et al. [13] defined pentagonal controlled fuzzy metric spaces (PCFMSs) and fuzzy controlled hexagonal metric spaces (FCHMSs) and extended the Banach contraction concept to PCFMSs.

In 2004, Park [14] introduced the concept of intuitionistic fuzzy metric spaces (IFMSs) and discussed the topological structure. Konwar [15] proposed the notion of intuitionistic fuzzy b-metric spaces (IFbMSs) as a generalization of IFMSs. Shatanawi et al. [16] used an E.A property and the common E.A property for coupled maps to obtain new results on generalized IFMSs. Gupta et al. [17] obtained some coupled fixed-point results on modified IFMSs and applied them to the integral-type contractions. Farheen et al. [18] introduced the concept of intuitionistic fuzzy double-controlled metric spaces (IFDCMSs) and proved some fixed-point results. Ishtiaq et al. [19] coined the concept of intuitionistic fuzzy double-controlled metric-like spaces and provided several non-trivial examples with their graphical views, for more related knowledge, see [20]. Younis and Abdou [21] presented novel fuzzy contractions and applications to engineering science. Ahmad et al. [22] presented the concept of bipolar b-metric spaces in the graph setting and related fixed point results.

We divide the paper into the six parts. In the first part, we present the introduction section. In the second part, we provide some basic and related definitions from the existing literature including CTN, CTCN, FTCMS, FEHBMS, CHFMS, and PCFMS. In the third part, we generalize the concepts of PCFMSs, FCHMSs and IFDCMSs and present the concepts of intuitionistic fuzzy pentagonal controlled metric spaces (IFPCMSs) and intuitionistic fuzzy controlled hexagonal metric spaces (IFCHMSs). We extend the Banach contraction principle in the setting of IFPCMSs. In the fourth part, we present an application to dynamic market equilibrium. In the fifth part, we provide an application to satellite web coupling. In the sixth part, we present the discussion and conclusion.

## 2. Preliminaries

This section contains some definitions from the existing literature that are useful for main section.

**Definition 2.1** [3] A binary operation *: [0, 1] × [0, 1] → [0, 1] is a CTN if it verifies the below axioms:

1. *τ* * *θ* = *θ* * *τ*, (∀) *τ*, *θ* ∈ [0, 1];
* is continuous;
3. *τ* * 1 = *τ*, (∀) *τ* ∈ [0, 1];
4. (*τ* * *θ*) * *ρ* = *τ* * (*θ* * *ρ*), (∀) *τ*, *θ*, *ρ* ∈ [0, 1];
5. if *τ* ≤ *ρ* and *θ* ≤ *σ*, with *τ*, *θ*, *ρ*, *σ* ∈ [0, 1], then *τ* * *θ* ≤ *ρ* * *σ*.

**Definition 2.2** [3] A binary operation Δ: [0, 1] × [0, 1] → [0, 1] is a CTCN if it verifies the below axioms:

1. *τ*Δ*θ* = *θ* Δ *τ*, (∀) *τ*, *θ* ∈ [0, 1];
2. Δ is continuous;
3. *τ*Δ0 = *τ*, (∀) *τ* ∈ [0, 1];
4. (*τ*Δ*θ*) Δ *ρ* = *τ*Δ(*θ*Δ*ρ*), (∀) *τ*, *θ*, *ρ* ∈ [0, 1];
5. if *τ* ≤ *ρ* and *θ* ≤ *σ*, with *τ*, *θ*, *ρ*, *σ* ∈ [0, 1], then *τ*Δ*θ* ≤ *ρ*Δ*σ*.

**Definition 2.3** [11] Let *X* be a non-empty set. A 3-tuple (*X*, *N*_*t*_,*,) is called a FTCMS if * is a CTN, *N*_*t*_ is a FS on *X* × *X* ×[0,∞) and *Q*, *W*, *E*: *X* × *X→*[1,∞) are non-comparable functions, satisfies the below conditions for all ϰ,d,e,κ∈X, ϰ≠e,e≠κ,κ≠d and *α*, *β*, *γ* > 0:

S1. $N_{t}\left(\varkappa ,d,0\right)=0;$
S2. ${\;N}_{t}\left(\varkappa ,d,\alpha \right)=1$ iff ϰ=d;
S3. ${\;N}_{t}\left(\varkappa ,d,\alpha \right)=N_{t}\left(d,\varkappa ,\alpha \right);$
S4. ${\;N}_{t}\left(\varkappa ,d,\alpha +\beta +\gamma \right)\geq N_{t}\left(\varkappa ,e,\frac{\alpha }{Q\left(\varkappa ,e\right)}\right)*N_{t}\left(e,\kappa ,\frac{\beta }{W\left(e,\kappa \right)}\right)*N_{t}\left(\kappa ,g,\frac{\gamma }{E\left(\kappa ,d\right)}\right);$
S5. *N*_*t*_(*ϰ*, *d*,.): (0, ∞) → [0,1] is left continuous and ${\text{lim}}_{\alpha \to \infty }\;N_{t}\left(\varkappa ,d,\alpha \right)=1.$

**Definition 2.4** [12] Let *X* be a non-empty set. A 3-tuple (*X*, *N*_*l*_, *) is called a FEHBMS, if * is a CTN, *N*_*l*_ is a FS on *X* × *X* ×[0,∞) and *Q*: *X × X* → [1, ∞) is a function, satisfies the below conditions for all ϰ,d,e,κ,g,ϖ∈X, ϰ≠e,e≠κ,κ≠g,g≠ϖ,ϖ≠d and *α*, *β*, *γ*, *δ*, *w* > 0:

F1. $N_{l}\left(\varkappa ,d,0\right)=0;$
F2. $N_{l}\left(\varkappa ,d,\alpha \right)=1$ iff ϰ=d;
F3. $N_{l}\left(\varkappa ,d,\alpha \right)=N_{l}\left(d,\varkappa ,\alpha \right);$
F4. $N_{l}\left(\varkappa ,d,Q\left(\varkappa ,d\right)(\alpha +\beta +\gamma +\delta +w)\right)\geq N_{l}\left(\varkappa ,e,\alpha \right)*N_{l}\left(e,\kappa ,\beta \right)*L\left(\kappa ,g,\gamma \right)*N_{l}\left(g,\varpi ,\delta \right)*N_{l}\left(\varpi ,d,w\right);$
F5. *N*_*l*_(*ϰ*, *d*,.): (0, ∞) → [0,1] is left continuous.

**Definition 2.5** [13] Let *X* be a non-empty set. A 3-tuple (*X*, *N*_*h*_, *) is called a CHFMS if * is a CTN, *N*_*h*,_ is a FS on *X* × *X* ×[0, ∞) and *Q*: *X × X* → [1, ∞) be a function, satisfies the below conditions for all ϰ,d,e,κ,g,ϖ∈X, ϰ≠e,e≠κ,κ≠g,g≠ϖ,ϖ≠d and *α*, *β*, *γ*, *δ*, *w* > 0:

T1. $N_{h}\left(\varkappa ,d,0\right)=0;$
T2. $N_{h}\left(\varkappa ,d,\alpha \right)=1$ iff ϰ=d;
T3. $N_{h}\left(\varkappa ,d,\alpha \right)=N_{h}\left(d,\varkappa ,\alpha \right);$
T4. $N_{h}\left(\varkappa ,d,\alpha +\beta +\gamma +\delta +w\right)\geq N_{h}\left(\varkappa ,e,\frac{\alpha }{Q\left(\varkappa ,e\right)}\right)*N_{h}\left(e,\kappa ,\frac{\beta }{Q\left(e,\kappa \right)}\right)*N_{h}\left(\kappa ,g,\frac{\gamma }{Q\left(\kappa ,g\right)}\right)*N_{h}\left(g,\varpi ,\frac{\delta }{Q\left(g,\varpi \right)}\right)*N_{h}\left(\varpi ,d,\frac{w}{Q\left(\varpi ,d\right)}\right);$
T5. *N*_*h*_(*ϰ*, *d*,.): (0, ∞) → [0,1] is left continuous and ${\text{lim}}_{\alpha \to \infty }\;N_{h}\left(\varkappa ,d,\alpha \right)=1.$

**Definition 2.6** [13] Let *X* be a non-empty set. A 3-tuple (*X*, *N*_*p*_, *) is called a PCFMS if * is a CTN, *N*_*p*_ is a FS on *X* × *X* ×[0, ∞), and *Q*, *W*, *E*, *R*, *T*: *X* × *X →*[1, ∞) are five non-comparable functions, satisfies the below conditions for all ϰ,d,e,κ,g,ϖ∈X, ϰ≠e,e≠κ,κ≠g,g≠ϖ,ϖ≠d and *α*, *β*, *γ*, *δ*, *w* > 0:

A1. $N_{p}\left(\varkappa ,d,0\right)=0;$
A2. $N_{p}\left(\varkappa ,d,\alpha \right)=1$ iff ϰ=d;
A3. $N_{p}\left(\varkappa ,d,\alpha \right)=N_{p}\left(d,\varkappa ,\alpha \right);$
A4. $N_{p}\left(\varkappa ,d,\alpha +\beta +\gamma +\delta +w\right)\geq N_{p}\left(\varkappa ,e,\frac{\alpha }{Q\left(\varkappa ,e\right)}\right)*N_{p}\left(e,\kappa ,\frac{\beta }{W\left(e,\kappa \right)}\right)*N_{p}\left(\kappa ,g,\frac{\gamma }{E\left(\kappa ,g\right)}\right)*N_{p}\left(g,\varpi ,\frac{\delta }{R\left(g,\varpi \right)}\right)*N_{p}\left(\varpi ,d,\frac{w}{T\left(\varpi ,d\right)}\right);$
A5. *N*_*p*_(*ϰ*, *d*,.): (0, ∞) → [0,1] is left continuous and ${\text{lim}}_{\alpha \to \infty }\;N_{p}\left(\varkappa ,d,\alpha \right)=1.$

## 3. Main results

In this section, we introduce the definitions of IFTCMS, IFCHMS and IFPCMS and provide fixed point theorems.

**Definition 3.1** Let *X* be a non-empty set. A 5-tuple (*X*, *N*_*t*_, *M*_*t*_, *, Δ) is called an IFTCMS if * is a CTN, Δ is a CTCN, *N*_*t*_, *M*_*t*_ are FSs on *X* × *X* × [0, ∞) and *Q*, *W*, *E*: *X* × *X →*[1, ∞) are non-comparable functions, satisfies the below conditions for all ϰ,d,e,κ∈X, ϰ≠e,e≠κ,κ≠d and *α*, *β*, *γ* > 0:

S1. ${\;N}_{t}\left(\varkappa ,d,\alpha \right)+{\;M}_{t}\left(\varkappa ,d,\alpha \right)\leq 1;$
S2. $N_{t}\left(\varkappa ,d,0\right)=0;$
S3. ${\;N}_{t}\left(\varkappa ,d,\alpha \right)=1$ iff ϰ=d;
S4. ${\;N}_{t}\left(\varkappa ,d,\alpha \right)=N_{t}\left(d,\varkappa ,\alpha \right);$
S5. $N_{t}\left(\varkappa ,d,\alpha +\beta +\gamma \right)\geq N_{t}\left(\varkappa ,e,\frac{\alpha }{Q\left(\varkappa ,e\right)}\right)*N_{t}\left(e,\kappa ,\frac{\beta }{W\left(e,\kappa \right)}\right)*N_{t}\left(\kappa ,g,\frac{\gamma }{E\left(\kappa ,d\right)}\right);$
S6. *N*_*t*_(*ϰ*, *d*,.): (0, ∞) → [0,1] is left continuous and ${\text{lim}}_{\alpha \to \infty }\;N_{t}\left(\varkappa ,d,\alpha \right)=1;$
S7. $M_{t}\left(\varkappa ,d,0\right)=0;$
S8. ${\;M}_{t}\left(\varkappa ,d,\alpha \right)=1$ iff ϰ=d;
S9. ${\;M}_{t}\left(\varkappa ,d,\alpha \right)=M_{t}\left(d,\varkappa ,\alpha \right);$
S10. $M_{t}\left(\varkappa ,d,\alpha +\beta +\gamma \right)\leq M_{t}\left(\varkappa ,e,\frac{\alpha }{Q\left(\varkappa ,e\right)}\right)\Delta \;M_{t}\left(e,\kappa ,\frac{\beta }{W\left(e,\kappa \right)}\right)\Delta \;M_{t}\left(\kappa ,g,\frac{\gamma }{E\left(\kappa ,d\right)}\right);$
S11. *M*_*t*_(*ϰ*, *d*,.): (0, ∞) → [0,1] is left continuous and ${\text{lim}}_{\alpha \to \infty }\;M_{t}\left(\varkappa ,d,\alpha \right)=0.$

**Example 3.1** Let *X* = [0,1]. Define *N*_*t*_, *M*_*t*_: *X* × *X* × [0,∞) →[0,1] as

$$N_{t}\left(\varkappa ,d,\alpha \right)=\frac{\alpha }{\alpha +\text{max}\;\left\{\varkappa ,d\right\}}\;for\;all\;\alpha >0,$$

$$M_{t}\left(\varkappa ,d,\alpha \right)=\frac{\text{max}\;\left\{\varkappa ,d\right\}}{\alpha +\text{max}\;\left\{\varkappa ,d\right\}}\;for\;all\;\alpha >0,$$

with the CTN * such that *α*_1_ * *α*_2_ = *α*_1_*α*_2_, and CTCN Δ such that *α*_1_ Δ *α*_2_ = max{*α*_1_, *α*_2_}. Then, (*X*, *N*_*t*_, *M*_*t*_,*, Δ) is an IFPCMS with non-comparable control functions

$$Q\left(\varkappa ,d\right)=1+\varkappa +d,\;W\left(\varkappa ,d\right)=1+\varkappa^{2}+d^{2},\;and\;E\left(\varkappa ,d\right)=1+\frac{\varkappa }{d}.$$

**Definition 3.2:** Let *X* be a non-empty set. A 5-tuple (*X*, *H*, *N*,*,Δ) is an IFEHbMS if * is a CTN, Δ is a CTCN, *H*, *N* are FSs on *X* × *X* × [0, ∞) and *Q*: *X* × *X →* [1, ∞), satisfies the below conditions for all ϰ,d,e,κ,g,ϖ∈X, ϰ≠e,e≠κ,κ≠g,g≠ϖ,ϖ≠d and *α*, *β*, *γ*, *δ*, *w* > 0:

C1. $H\left(\varkappa ,d,\alpha \right)+N\left(\varkappa ,d,\alpha \right)\leq 1;$
C2. $H\left(\varkappa ,d,0\right)=0;$
C3. $H\left(\varkappa ,d,\alpha \right)=1$ iff ϰ=d;
C4. $H\left(\varkappa ,d,\alpha \right)=H\left(d,\varkappa ,\alpha \right);$
C5. $H\left(\varkappa ,d,Q\left(\varkappa ,d\right)\left(\alpha +\beta +\gamma +\delta +w\right)\right)\geq H\left(\varkappa ,e,\alpha \right)*H\left(e,\kappa ,\beta \right)*H\left(\kappa ,g,\gamma \right)*\;H\left(g,\varpi ,\delta \right)*H\left(\varpi ,d,w\right);$
C6. *H*(*ϰ*, *d*,.): (0, ∞) → [0,1] is left continuous and ${\text{lim}}_{\alpha \to \infty }\;H\left(\varkappa ,d,\alpha \right)=1;$
C7. $N\left(\varkappa ,d,0\right)=1;$
C8. $N\left(\varkappa ,d,\alpha \right)=0$ iff ϰ=d;
C9. $N\left(\varkappa ,d,\alpha \right)=N\left(d,\varkappa ,\alpha \right);$
C10. $N\left(\varkappa ,d,Q\left(\varkappa ,d\right)\left(\alpha +\beta +\gamma +\delta +w\right)\right)\leq N\left(\varkappa ,e,\alpha \right)\Delta \;N\left(e,\kappa ,\beta \right)\Delta \;N\left(\kappa ,g,\gamma \right)\Delta \;N\left(g,\varpi ,\delta \right)\Delta N\left(\varpi ,d,w\right);$
C11. *N*(*ϰ*, *d*,.): (0, ∞) → [0,1] is left continuous and ${\text{lim}}_{\alpha \to \infty }\;N\left(\varkappa ,d,\alpha \right)=0.$

**Definition 3.3:** Let *X* be a non-empty set. A 5-tuple (*X*, *H*, *N*, *, Δ) is an IFCHMS if * is a CTN, Δ is a CTCN, *H*, *N* are FSs on *X × X ×* [0, ∞) and *Q*: *X* × *X →* [1, ∞) satisfies the below conditions for all ϰ,d,e,κ,g,ϖ∈X, ϰ≠e,e≠κ,κ≠g,g≠ϖ,ϖ≠d and *α*, *β*, *γ*, *δ*, *w* > 0:

C12. $H\left(\varkappa ,d,\alpha \right)+N\left(\varkappa ,d,\alpha \right)\leq 1;$
C13. $H\left(\varkappa ,d,0\right)=0;$
C14. $H\left(\varkappa ,d,\alpha \right)=1$ iff ϰ=d;
C15. $H\left(\varkappa ,d,\alpha \right)=H\left(d,\varkappa ,\alpha \right);$
C16. $H\left(\varkappa ,d,\alpha +\beta +\gamma +\delta +w\right)\geq H\left(\varkappa ,e,\frac{\alpha }{Q\left(\varkappa ,e\right)}\right)*H\left(e,\kappa ,\frac{\beta }{Q\left(e,\kappa \right)}\right)*H\left(\kappa ,g,\frac{\gamma }{Q\left(\kappa ,g\right)}\right)*\;H\left(g,\varpi ,\frac{\delta }{Q\left(g,\varpi \right)}\right)*H\left(\varpi ,d,\frac{w}{Q\left(\varpi ,d\right)}\right);$
C17. *H*(*ϰ*, *d*,.):(0, ∞) → [0,1] is left continuous and ${\text{lim}}_{\alpha \to \infty }\;H\left(\varkappa ,d,\alpha \right)=1;$
C18. $N\left(\varkappa ,d,0\right)=1;$
C19. $N\left(\varkappa ,d,\alpha \right)=0$ iff ϰ=d;
C20. $N\left(\varkappa ,d,\alpha \right)=N\left(d,\varkappa ,\alpha \right);$
C21. $N\left(\varkappa ,d,\alpha +\beta +\gamma +\delta +w\right)\leq N\left(\varkappa ,e,\frac{\alpha }{Q\left(\varkappa ,e\right)}\right)\Delta \;N\left(e,\kappa ,\frac{\beta }{Q\left(e,\kappa \right)}\right)\Delta \;N\left(\kappa ,g,\frac{\gamma }{Q\left(\kappa ,g\right)}\right)\Delta \;N\left(g,\varpi ,\frac{\delta }{Q\left(g,\varpi \right)}\right)\Delta N\left(\varpi ,d,\frac{w}{Q\left(\varpi ,d\right)}\right);$
C22. *N*(*ϰ*, *d*,.): (0, ∞) → [0,1] is left continuous and ${\text{lim}}_{\alpha \to \infty }\;N\left(\varkappa ,d,\alpha \right)=0.$

**Example 3.2:** Let *X* = {1,2,3,4,5,6}. Define *H*, *N*: *X* × *X* × [0,∞) → [0,1] as

$$H\left(\varkappa ,d,\alpha \right)={\left[e^{\frac{{\left|\varkappa -d\right|}^{2}}{\alpha }}\right]}^{-1}\;for\;all\;\alpha >0,$$

$$N\left(\varkappa ,d,\alpha \right)=1-{\left[e^{\frac{{\left|\varkappa -d\right|}^{2}}{\alpha }}\right]}^{-1}\;for\;all\;\alpha >0,$$

with the CTN * such that *α*_1_ * *α*_2_ = *α*_1_*α*_2_, and CTCN Δ such that *α*_1_ Δ *α*_2_ = max{*α*_1,_ *α*_2_}. Then, (*X*, *H*, *N*, *, Δ) is an IFCHMS with a control function $Q\left(\varkappa ,d\right)=1+\varkappa +d.$

**Definition 3.4:** Let *X* be a non-empty set. A 5-tuple (*X*, *M*, *N*,*, Δ) is an IFPCMS if * is a CTN, Δ is a CTCN, *M* and *N* are FSs on *X* × *X* × [0, ∞) and *Q*, *W*, *E*, *R*, *T*: *X* × *X →*[1, ∞) are five non-comparable functions, satisfies the below conditions for all ϰ,d,e,κ,g,ϖ∈X, ϰ≠e,e≠κ,κ≠g,g≠ϖ,ϖ≠d and *α*, *β*, *γ*, *δ*, *w* > 0:

IFP1. $M\left(\varkappa ,d,\alpha \right)+N\left(\varkappa ,d,\alpha \right)\leq 1;$
IFP2. $M\left(\varkappa ,d,0\right)=0;$
IFP3. $M\left(\varkappa ,d,\alpha \right)=1$ iff ϰ=d;
IFP4. $M\left(\varkappa ,d,\alpha \right)=M\left(d,\varkappa ,\alpha \right);$
IFP5. $(M\left(\varkappa ,d,\alpha +\beta +\gamma +\delta +w\right)\geq M\left(\varkappa ,e,\frac{\alpha }{Q\left(\varkappa ,e\right)}\right)*M\left(e,\kappa ,\frac{\beta }{W\left(e,\kappa \right)}\right)*M\left(\kappa ,g,\frac{\gamma }{E\left(\kappa ,g\right)}\right)*M\left(g,\varpi ,\frac{\delta }{R\left(g,\varpi \right)}\right)*M\left(\varpi ,d,\frac{w}{T\left(\varpi ,d\right)}\right);$
IFP6. *M*(*ϰ*, *d*,.): (0, ∞) → [0,1] is left continuous and ${\text{lim}}_{\alpha \to \infty }\;M\left(\varkappa ,d,\alpha \right)=1;$
IFP7. $N\left(\varkappa ,d,0\right)=1;$
IFP8. $N\left(\varkappa ,d,\alpha \right)=0$ iff ϰ=d;
IFP9. $N\left(\varkappa ,d,\alpha \right)=N\left(d,\varkappa ,\alpha \right);$
IFP10. $N\left(\varkappa ,d,\alpha +\beta +\gamma +\delta +w\right)\leq N\left(\varkappa ,e,\frac{\alpha }{Q\left(\varkappa ,e\right)}\right)\Delta \;N\left(e,\kappa ,\frac{\beta }{W\left(e,\kappa \right)}\right)\Delta \;N\left(\kappa ,g,\frac{\gamma }{E\left(\kappa ,g\right)}\right)\Delta \;N\left(g,\varpi ,\frac{\delta }{R\left(g,\varpi \right)}\right)\Delta \;N\left(\varpi ,d,\frac{w}{T\left(\varpi ,d\right)}\right);$
IFP11. *N*(*ϰ*, *d*,.): (0, ∞) → [0,1] is left continuous, ${\text{lim}}_{\alpha \to \infty }\;N\left(\varkappa ,d,\alpha \right)=0.$

**Example 3.3** Let *X* = [0, 1]. Define *M*, *N*: *X* × *X* × [0, ∞) → [0, 1] as

$$M\left(\varkappa ,d,\alpha \right)=\frac{\alpha }{\alpha +{\left|\varkappa -d\right|}^{6}}\;for\;all\;\alpha >0,$$

$$N\left(\varkappa ,d,\alpha \right)=\frac{{\left|\varkappa -d\right|}^{6}}{\alpha +{\left|\varkappa -d\right|}^{6}}\;for\;all\;\alpha >0,$$

with the CTN * such that *α*_1_ * *α*_2_ = *α*_1_*α*_2_, and CTCN Δ such that *α*_1_ Δ *α*_2_ = max{*α*_1,_ *α*_2_}. Then, (*X*, *H*, *N*, *, Δ) is an IFPCMS with non-comparable control functions $Q\left(\varkappa ,d\right)=1+\varkappa +d,$

$$W\left(\varkappa ,d\right)=1+\varkappa^{2}+d^{2},\;E\left(\varkappa ,d\right)=1+\frac{\varkappa }{d},\;R\left(\varkappa ,d\right)=1+\frac{d}{\varkappa },\;and\;T\left(\varkappa ,d\right)=1+\varkappa^{2}+d.$$

**Remark 3.1** From the definition of IFPCMS,

1. If we take $W\left(e,\kappa \right)=Q\left(e,\kappa \right),\;E\left(\kappa ,g\right)=Q\left(\kappa ,g\right),\;R\left(g,\varpi \right)=Q\left(g,\varpi \right),T\left(\varpi ,d\right)=Q\left(\varpi ,d\right),$ then it will become the definition of IFCHMS.
2. If we take $Q\left(\varkappa ,e\right)=W\left(e,\kappa \right)=E\left(\kappa ,g\right)=R\left(g,\varpi \right)=T\left(\varpi ,d\right)=b\left(\varkappa ,d\right),$ then it will become the definition of IFEHbMS.
3. If g=ϖ=d and *γ* + *δ* +*w* = *r*′, then it will become IFTCMS.
4. If κ=g=ϖ=d and *β* + *γ* + *δ* + *w* = *t*′, then it will become IFDCMS in [18].
5. If κ=g=ϖ=d, *β* + *γ* + *δ* + *w* = *t*′, and *W*(*e*, *κ*) = *E*(*κ*, *g*) = *b* ≥ 1 then it will become IFbMS in [15].
6. If κ=g=ϖ=d, *β* + *γ* + *δ* + *w* = *t*′, and *W*(*e*, *κ*) = *E*(*κ*, *g*) = 1 then it will become IFMS in [14].
7. Every PCFMS is an IFPCMS of the form (*X*, *M*, 1 − *M*, *, Δ), if we take $Z\Delta d=1-\left(\left(1-\varkappa \right)*\left(1-d\right)\right).$

**Definition 3.5** Let (*X*, *M*, *N*, *, Δ) is a IPCFMS and $\left\{\varkappa_{n}\right\}$ be a sequence in *X*, then $\left\{\varkappa_{n}\right\}$ is called:

**(a)** a convergent, if there exists ϰ∈X such that
$$\underset{n\to \infty }{\text{lim}}\;M\left(\varkappa_{n},\;\varkappa ,\;\alpha \right)=1and\;\underset{n\to \infty }{\text{lim}}\;N\left(\varkappa_{n},\;\varkappa ,\;\alpha \right)=0,\;for\;all\;\alpha >0,$$
**(b)** a Cauchy, if and only if for each *ω* > 0, *α* > 0, there exists $n_{0}\in N$ such that
$$M\left(\varkappa_{n},\;\varkappa_{m},\;\alpha \right)\geq 1-\omega ,\;and\;N\left(\varkappa_{n},\;\varkappa_{m},\;\alpha \right)\leq \omega ,\;for\;all\;n,m\geq n_{0},$$
**(c)** if every Cauchy sequence is convergent in *X*, then (*X*, *M*, *N*, *, Δ) is a complete IFPCMS.

**Definition 3.6** Let (*X*, *M*, *N*, *, Δ) is an IFPCMS, then we define an open ball $B\left(\varkappa ,r,\alpha \right)$ with centre ϰ, radius *r*, 0 < *r* < 1 and *α* > 0 as follows:

$$B\left(\varkappa ,r,\alpha \right)=\left\{d\in X:M\left(\varkappa ,d,\alpha \right)>1-r\right\},$$

$$B\left(\varkappa ,r,\alpha \right)=\left\{d\in X:N\left(\varkappa ,d,\alpha \right)<r\right\},$$

and the topology that corresponds to it is defined as

$$\tau_{p\varkappa }=\left\{D\subset X:B\left(\varkappa ,r,\alpha \right)\subset D\right\}.$$

**Theorem 3.1** Let (*X*, *M*, *N*,*,Δ) be a complete IFPCMS and *Q*, *W*, *E*, *R*, *T*:*X* × *X* → [1,∞) such that

$$\underset{\alpha \to \infty }{\lim}M\left(\varkappa ,d,\alpha \right)=1,\;\;\;\text{and}\;\underset{\alpha \to \infty }{\lim}N\left(\varkappa ,d,\alpha \right)=0,\;\text{for}\;\text{all}\;\alpha >0\;\text{and}\;\varkappa ,d\in X.$$ (1)

Let *F*: *X → X* be a mapping satisfying

$$M\left(F\varkappa ,Fd,q\alpha \right)\geq M\left(\varkappa ,d,\alpha \right),$$ (2)

and

$$N\left(F\varkappa ,Fd,q\alpha \right)\leq N\left(\varkappa ,d,\alpha \right)\;for\;all\;\alpha >0\;and\;\varkappa ,d\in X,$$ (3)

where 0 < *p <*1. Furthermore, if, for $\varkappa_{0}\in X\;and\;n,q\in \left\{1,2,3,\cdots \right\},$ It holds $b\left(\varkappa_{n},\varkappa_{n+q}\right)<\frac{1}{p}$ where $\varkappa_{n}=F^{n}\varkappa_{0},$ then *F* has a unique fixed point.

**Proof:** Let $\varkappa_{0}\in X$ and define a sequence $\left\{\varkappa_{n}\right\}$ by

$$\varkappa_{n}={F\varkappa }_{n-1}\;for\;all\;n\in \left\{1,2,3,\cdots \right\}.$$

Without loss of generality, assume that $\varkappa_{n}\neq \varkappa_{n+1}\;for\;all\;n\in \left\{0,1,2,3,\cdots \right\}.$ With the help of (2), and (3), we deduce

$$\begin{array}{c} M\left(\varkappa_{n},\varkappa_{n+1},\alpha \right)=M\left(F\varkappa_{n-1},F\varkappa_{n},\alpha \right)\\ \geq M\left(\varkappa_{n-1},\varkappa_{n},\frac{\alpha }{p}\right)\\ \vdots \\ \geq M\left(\varkappa_{0},\varkappa_{1},\frac{\alpha }{p^{n}}\right). \end{array}$$ (4)

Continuing on the same lines, we obtain

$$M\left(\varkappa_{n},\varkappa_{n+2},\alpha \right)\geq M\left(\varkappa_{0},\varkappa_{2},\frac{\alpha }{p^{n}}\right),$$ (5)

$$M\left(\varkappa_{n},\varkappa_{n+3},\alpha \right)\geq M\left(\varkappa_{0},\varkappa_{3},\frac{\alpha }{p^{n}}\right),$$ (6)

$$M\left(\varkappa_{n},\varkappa_{n+4},\alpha \right)\geq M\left(\varkappa_{0},\varkappa_{4},\frac{\alpha }{p^{n}}\right).$$ (7)

It implies, if *m* = 1,2,3,⋯,

$$M\left(\varkappa_{n},\varkappa_{n+4m+1},\alpha \right)\geq M\left(\varkappa_{0},\varkappa_{4m+1},\frac{\alpha }{p^{n}}\right),$$ (8)

$$M\left(\varkappa_{n},\varkappa_{n+4m+2},\alpha \right)\geq M\left(\varkappa_{0},\varkappa_{4m+2},\frac{\alpha }{p^{n}}\right),$$ (9)

$$M\left(\varkappa_{n},\varkappa_{n+4m+3},\alpha \right)\geq M\left(\varkappa_{0},\varkappa_{4m+3},\frac{\alpha }{p^{n}}\right),$$ (10)

$$M\left(\varkappa_{n},\varkappa_{n+4m+4},\alpha \right)\geq M\left(\varkappa_{0},\varkappa_{4m+4},\frac{\alpha }{p^{n}}\right),$$ (11)

and

$$\begin{array}{c} N\left(\varkappa_{n},\varkappa_{n+1},\alpha \right)=M\left(F\varkappa_{n-1},F\varkappa_{n},\alpha \right)\\ \leq N\left(\varkappa_{n-1},\varkappa_{n},\frac{\alpha }{p}\right)\\ \vdots \\ \leq N\left(\varkappa_{0},\varkappa_{1},\frac{\alpha }{p^{n}}\right). \end{array}$$ (12)

Continuing on the same lines, we obtain

$$N\left(\varkappa_{n},\varkappa_{n+2},\alpha \right)\leq N\left(\varkappa_{0},\varkappa_{2},\frac{\alpha }{p^{n}}\right),$$ (13)

$$N\left(\varkappa_{n},\varkappa_{n+3},\alpha \right)\leq N\left(\varkappa_{0},\varkappa_{3},\frac{\alpha }{p^{n}}\right),$$ (14)

$$N\left(\varkappa_{n},\varkappa_{n+4},\alpha \right)\leq N\left(\varkappa_{0},\varkappa_{4},\frac{\alpha }{p^{n}}\right).$$ (15)

It implies, if *m* = 1,2,3,⋯,

$$N\left(\varkappa_{n},\varkappa_{n+4m+1},\alpha \right)\leq N\left(\varkappa_{0},\varkappa_{4m+1},\frac{\alpha }{p^{n}}\right),$$ (16)

$$N\left(\varkappa_{n},\varkappa_{n+4m+2},\alpha \right)\leq N\left(\varkappa_{0},\varkappa_{4m+2},\frac{\alpha }{p^{n}}\right),$$ (17)

$$N\left(\varkappa_{n},\varkappa_{n+4m+3},\alpha \right)\leq N\left(\varkappa_{0},\varkappa_{4m+3},\frac{\alpha }{p^{n}}\right),$$ (18)

$$N\left(\varkappa_{n},\varkappa_{n+4m+4},\alpha \right)\leq N\left(\varkappa_{0},\varkappa_{4m+4},\frac{\alpha }{p^{n}}\right).$$ (19)

Expressing $\alpha =\frac{\alpha }{5}+\frac{\alpha }{5}+\frac{\alpha }{5}+\frac{\alpha }{5}+\frac{\alpha }{5}$ and by using (4), (12), (A2) and (A7), we obtain

$$\begin{array}{c} M\left(\varkappa_{0},\varkappa_{5},\alpha \right)\geq M\left(\varkappa_{0},\varkappa_{1},\frac{\alpha }{5Q\left(\varkappa_{0},\varkappa_{1}\right)}\right)\\ {}*M\left(\varkappa_{0},\varkappa_{1},\frac{\alpha }{5pW\left(\varkappa_{1},\varkappa_{2}\right)}\right)*M\left(\varkappa_{0},\varkappa_{1},\frac{\alpha }{5p^{2}E\left(\varkappa_{2},\varkappa_{3}\right)}\right)\\ {}*M\left(\varkappa_{0},\varkappa_{1},\frac{\alpha }{5p^{3}R\left(\varkappa_{3},\varkappa_{4}\right)}\right)*M\left(\varkappa_{0},\varkappa_{1},\frac{\alpha }{5p^{4}T\left(\varkappa_{4},\varkappa_{5}\right)}\right), \end{array}$$

and

$$\begin{array}{l} N\left(\varkappa_{0},\varkappa_{5},\alpha \right)\leq N\left(\varkappa_{0},\varkappa_{1},\frac{\alpha }{5Q\left(\varkappa_{0},\varkappa_{1}\right)}\right)\Delta \;N\left(\varkappa_{0},\varkappa_{1},\frac{\alpha }{5pW\left(\varkappa_{1},\varkappa_{2}\right)}\right)\\ \Delta \;N\left(\varkappa_{0},\varkappa_{1},\frac{\alpha }{5p^{2}E\left(\varkappa_{2},\varkappa_{3}\right)}\right)\Delta \;N\left(\varkappa_{0},\varkappa_{1},\frac{\alpha }{5p^{3}R\left(\varkappa_{3},\varkappa_{4}\right)}\right)\Delta \;N\left(\varkappa_{0},\varkappa_{1},\frac{\alpha }{5p^{4}T\left(\varkappa_{4},\varkappa_{5}\right)}\right). \end{array}$$

In similar manner, we can deduce

$$\begin{array}{c} M\left(\varkappa_{0},\varkappa_{9},\alpha \right)\geq M\left(\varkappa_{0},\varkappa_{1},\frac{\alpha }{5Q\left(\varkappa_{0},\varkappa_{1}\right)}\right)*M\left(\varkappa_{0},\varkappa_{1},\frac{\alpha }{5pW\left(\varkappa_{1},\varkappa_{2}\right)}\right)\\ {}*M\left(\varkappa_{0},\varkappa_{1},\frac{\alpha }{5p^{2}E\left(\varkappa_{2},\varkappa_{3}\right)}\right)*M\left(\varkappa_{0},\varkappa_{1},\frac{\alpha }{5p^{3}R\left(\varkappa_{3},\varkappa_{4}\right)}\right)\\ {}*M\left(\varkappa_{0},\varkappa_{1},\frac{\alpha }{{\left(5\right)}^{2}p^{4}T\left(\varkappa_{4},\varkappa_{9}\right)Q\left(\varkappa_{4},\varkappa_{5}\right)}\right)*M\left(\varkappa_{0},\varkappa_{1},\frac{\alpha }{{\left(5\right)}^{2}p^{5}T\left(\varkappa_{4},\varkappa_{9}\right)W\left(\varkappa_{5},\varkappa_{6}\right)}\right)\\ {}*M\left(\varkappa_{0},\varkappa_{1},\frac{\alpha }{{\left(5\right)}^{2}p^{6}T\left(\varkappa_{4},\varkappa_{9}\right)E\left(\varkappa_{6},\varkappa_{7}\right)}\right)*M\left(\varkappa_{0},\varkappa_{1},\frac{\alpha }{{\left(5\right)}^{2}p^{7}T\left(\varkappa_{4},\varkappa_{9}\right)R\left(\varkappa_{7},\varkappa_{8}\right)}\right)\\ {}*M\left(\varkappa_{0},\varkappa_{1},\frac{\alpha }{{\left(5\right)}^{2}p^{8}T\left(\varkappa_{4},\varkappa_{9}\right)T\left(\varkappa_{8},\varkappa_{9}\right)}\right), \end{array}$$

and

$$\begin{array}{c} N\left(\varkappa_{0},\varkappa_{9},\alpha \right)\leq N\left(\varkappa_{0},\varkappa_{1},\frac{\alpha }{5Q\left(\varkappa_{0},\varkappa_{1}\right)}\right)\Delta \;N\left(\varkappa_{0},\varkappa_{1},\frac{\alpha }{5pW\left(\varkappa_{1},\varkappa_{2}\right)}\right)\Delta \;N\left(\varkappa_{0},\varkappa_{1},\frac{\alpha }{5p^{2}E\left(\varkappa_{2},\varkappa_{3}\right)}\right)\\ \Delta \;N\left(\varkappa_{0},\varkappa_{1},\frac{\alpha }{5p^{3}R\left(\varkappa_{3},\varkappa_{4}\right)}\right)\Delta \;N\left(\varkappa_{0},\varkappa_{1},\frac{\alpha }{{\left(5\right)}^{2}p^{4}T\left(\varkappa_{4},\varkappa_{9}\right)Q\left(\varkappa_{4},\varkappa_{5}\right)}\right)\\ \Delta \;N\left(\varkappa_{0},\varkappa_{1},\frac{\alpha }{{\left(5\right)}^{2}p^{5}T\left(\varkappa_{4},\varkappa_{9}\right)W\left(\varkappa_{5},\varkappa_{6}\right)}\right)\Delta \;N\left(\varkappa_{0},\varkappa_{1},\frac{\alpha }{{\left(5\right)}^{2}p^{6}T\left(\varkappa_{4},\varkappa_{9}\right)E\left(\varkappa_{6},\varkappa_{7}\right)}\right)\\ \Delta \;N\left(\varkappa_{0},\varkappa_{1},\frac{\alpha }{{\left(5\right)}^{2}p^{7}T\left(\varkappa_{4},\varkappa_{9}\right)R\left(\varkappa_{7},\varkappa_{8}\right)}\right)\Delta \;N\left(\varkappa_{0},\varkappa_{1},\frac{\alpha }{{\left(5\right)}^{2}p^{8}T\left(\varkappa_{4},\varkappa_{9}\right)T\left(\varkappa_{8},\varkappa_{9}\right)}\right). \end{array}$$

We obtain for each *m* = 1,2,3,⋯,

$$\begin{array}{c} M\left(\varkappa_{0},\varkappa_{4m+1},\alpha \right)\geq M\left(\varkappa_{0},\varkappa_{1},\frac{\alpha }{5Q\left(\varkappa_{0},\varkappa_{1}\right)}\right)*M\left(\varkappa_{1},\varkappa_{2},\frac{\alpha }{5W\left(\varkappa_{1},\varkappa_{2}\right)}\right)*M\left(\varkappa_{2},\varkappa_{3},\frac{\alpha }{5E\left(\varkappa_{2},\varkappa_{3}\right)}\right)\\ {}*M\left(\varkappa_{3},\varkappa_{4},\frac{\alpha }{5R\left(\varkappa_{3},\varkappa_{4}\right)}\right)*M\left(\varkappa_{4},\varkappa_{4m+1},\frac{\alpha }{5T\left(\varkappa_{4},\varkappa_{4m+1}\right)}\right) \end{array}$$

$$\begin{array}{c} \geq M\left(\varkappa_{0},\varkappa_{1},\frac{\alpha }{5Q\left(\varkappa_{0},\varkappa_{1}\right)}\right)*M\left(\varkappa_{0},\varkappa_{1},\frac{\alpha }{5pW\left(\varkappa_{1},\varkappa_{2}\right)}\right)*M\left(\varkappa_{0},\varkappa_{1},\frac{\alpha }{5p^{2}E\left(\varkappa_{2},\varkappa_{3}\right)}\right)\\ {}*M\left(\varkappa_{0},\varkappa_{1},\frac{\alpha }{5p^{3}R\left(\varkappa_{3},\varkappa_{4}\right)}\right)*M\left(\varkappa_{0},\varkappa_{1},\frac{\alpha }{{\left(5\right)}^{2}p^{4}T\left(\varkappa_{4},\varkappa_{4m+1}\right)Q\left(\varkappa_{4},\varkappa_{5}\right)}\right)\\ {}*M\left(\varkappa_{0},\varkappa_{1},\frac{\alpha }{{\left(5\right)}^{2}p^{5}T\left(\varkappa_{4},\varkappa_{4m+1}\right)W\left(\varkappa_{5},\varkappa_{6}\right)}\right)*M\left(\varkappa_{0},\varkappa_{1},\frac{\alpha }{{\left(5\right)}^{2}p^{6}T\left(\varkappa_{4},\varkappa_{4m+1}\right)E\left(\varkappa_{6},\varkappa_{7}\right)}\right)\\ {}*M\left(\varkappa_{0},\varkappa_{1},\frac{\alpha }{{\left(5\right)}^{2}p^{7}T\left(\varkappa_{4},\varkappa_{4m+1}\right)R\left(\varkappa_{7},\varkappa_{8}\right)}\right)*M\left(\varkappa_{0},\varkappa_{1},\frac{\alpha }{{\left(5\right)}^{3}p^{8}T\left(\varkappa_{4},\varkappa_{4m+1}\right)T\left(\varkappa_{8},\varkappa_{4m+1}\right)Q\left(\varkappa_{8},\varkappa_{9}\right)}\right)\\ {}*M\left(\varkappa_{0},\varkappa_{1},\frac{\alpha }{{\left(5\right)}^{3}p^{9}T\left(\varkappa_{4},\varkappa_{4m+1}\right)T\left(\varkappa_{8},\varkappa_{4m+1}\right)W\left(\varkappa_{9},\varkappa_{10}\right)}\right)*M\left(\varkappa_{0},\varkappa_{1},\frac{\alpha }{{\left(5\right)}^{3}p^{10}T\left(\varkappa_{4},\varkappa_{4m+1}\right)T\left(\varkappa_{8},\varkappa_{4m+1}\right)E\left(\varkappa_{10},\varkappa_{11}\right)}\right)\\ {}*M\left(\varkappa_{0},\varkappa_{1},\frac{\alpha }{{\left(5\right)}^{3}p^{11}T\left(\varkappa_{4},\varkappa_{4m+1}\right)T\left(\varkappa_{8},\varkappa_{4m+1}\right)R\left(\varkappa_{11},\varkappa_{12}\right)}\right)\\ {}*M\left(\varkappa_{0},\varkappa_{1},\frac{\alpha }{{\left(5\right)}^{4}p^{12}T\left(\varkappa_{4},\varkappa_{4m+1}\right)T\left(\varkappa_{8},\varkappa_{4m+1}\right)T\left(\varkappa_{12},\varkappa_{4m+1}\right)Q\left(\varkappa_{12},\varkappa_{13}\right)}\right) \end{array}$$

$$\begin{array}{c} {}*\\ \vdots \\ {}*\\ M\left(\varkappa_{0},\varkappa_{1},\frac{\alpha }{{\left(5\right)}^{m}p^{4m}T\left(\varkappa_{4},\varkappa_{4m+1}\right)T\left(\varkappa_{8},\varkappa_{4m+1}\right)T\left(\varkappa_{12},\varkappa_{4m+1}\right)\cdots T\left(\varkappa_{4m-4},\varkappa_{4m+1}\right)T\left(\varkappa_{4m},\varkappa_{4m+1}\right)}\right), \end{array}$$

and

$$N\left(\varkappa_{0},\varkappa_{4m+1},\alpha \right)\leq N\left(\varkappa_{0},\varkappa_{1},\frac{\alpha }{5Q\left(\varkappa_{0},\varkappa_{1}\right)}\right)\Delta \;N\left(\varkappa_{1},\varkappa_{2},\frac{\alpha }{5W\left(\varkappa_{1},\varkappa_{2}\right)}\right)\Delta \;N\left(\varkappa_{2},\varkappa_{3},\frac{\alpha }{5E\left(\varkappa_{2},\varkappa_{3}\right)}\right)$$

$$\Delta \;N\left(\varkappa_{3},\varkappa_{4},\frac{\alpha }{5R\left(\varkappa_{3},\varkappa_{4}\right)}\right)\Delta \;N\left(\varkappa_{4},\varkappa_{4m+1},\frac{\alpha }{5T\left(\varkappa_{4},\varkappa_{4m+1}\right)}\right)$$

$$\leq N\left(\varkappa_{0},\varkappa_{1},\frac{\alpha }{5Q\left(\varkappa_{0},\varkappa_{1}\right)}\right)\Delta \;N\left(\varkappa_{0},\varkappa_{1},\frac{\alpha }{5pW\left(\varkappa_{1},\varkappa_{2}\right)}\right)\Delta \;N\left(\varkappa_{0},\varkappa_{1},\frac{\alpha }{5p^{2}E\left(\varkappa_{2},\varkappa_{3}\right)}\right)\Delta \;N\left(\varkappa_{0},\varkappa_{1},\frac{\alpha }{5p^{3}R\left(\varkappa_{3},\varkappa_{4}\right)}\right)$$

$$\Delta \;N\left(\varkappa_{0},\varkappa_{1},\frac{\alpha }{{\left(5\right)}^{2}p^{4}T\left(\varkappa_{4},\varkappa_{4m+1}\right)Q\left(\varkappa_{4},\varkappa_{5}\right)}\right)\Delta \;N\left(\varkappa_{0},\varkappa_{1},\frac{\alpha }{{\left(5\right)}^{2}p^{5}T\left(\varkappa_{4},\varkappa_{4m+1}\right)W\left(\varkappa_{5},\varkappa_{6}\right)}\right)$$

$$\Delta \;N\left(\varkappa_{0},\varkappa_{1},\frac{\alpha }{{\left(5\right)}^{2}p^{6}T\left(\varkappa_{4},\varkappa_{4m+1}\right)E\left(\varkappa_{6},\varkappa_{7}\right)}\right)\Delta \;N\left(\varkappa_{0},\varkappa_{1},\frac{\alpha }{{\left(5\right)}^{2}p^{7}T\left(\varkappa_{4},\varkappa_{4m+1}\right)R\left(\varkappa_{7},\varkappa_{8}\right)}\right)$$

$$\Delta \;N\left(\varkappa_{0},\varkappa_{1},\frac{\alpha }{{\left(5\right)}^{3}p^{8}T\left(\varkappa_{4},\varkappa_{4m+1}\right)T\left(\varkappa_{8},\varkappa_{4m+1}\right)Q\left(\varkappa_{8},\varkappa_{9}\right)}\right)$$

$$\Delta \;N\left(\varkappa_{0},\varkappa_{1},\frac{\alpha }{{\left(5\right)}^{3}p^{9}T\left(\varkappa_{4},\varkappa_{4m+1}\right)T\left(\varkappa_{8},\varkappa_{4m+1}\right)W\left(\varkappa_{9},\varkappa_{10}\right)}\right)$$

$$\Delta \;N\left(\varkappa_{0},\varkappa_{1},\frac{\alpha }{{\left(5\right)}^{3}p^{10}T\left(\varkappa_{4},\varkappa_{4m+1}\right)T\left(\varkappa_{8},\varkappa_{4m+1}\right)E\left(\varkappa_{10},\varkappa_{11}\right)}\right)$$

$$\Delta \;N\left(\varkappa_{0},\varkappa_{1},\frac{\alpha }{{\left(5\right)}^{3}p^{11}T\left(\varkappa_{4},\varkappa_{4m+1}\right)T\left(\varkappa_{8},\varkappa_{4m+1}\right)R\left(\varkappa_{11},\varkappa_{12}\right)}\right)$$

$$\Delta \;N\left(\varkappa_{0},\varkappa_{1},\frac{\alpha }{{\left(5\right)}^{4}p^{12}T\left(\varkappa_{4},\varkappa_{4m+1}\right)T\left(\varkappa_{8},\varkappa_{4m+1}\right)T\left(\varkappa_{12},\varkappa_{4m+1}\right)Q\left(\varkappa_{12},\varkappa_{13}\right)}\right)$$

$$\begin{array}{c} \Delta \\ \vdots \\ \Delta \\ N\left(\varkappa_{0},\varkappa_{1},\frac{\alpha }{{\left(5\right)}^{m}p^{4m}T\left(\varkappa_{4},\varkappa_{4m+1}\right)T\left(\varkappa_{8},\varkappa_{4m+1}\right)T\left(\varkappa_{12},\varkappa_{4m+1}\right)\cdots T\left(\varkappa_{4m-4},\varkappa_{4m+1}\right)T\left(\varkappa_{4m},\varkappa_{4m+1}\right)}\right). \end{array}$$

Now, using (8), and (16), we deduce that

$$\begin{array}{c} M\left(\varkappa_{n},\varkappa_{n+4m+1},\alpha \right)\geq M\left(\varkappa_{0},\varkappa_{4m+1},\frac{\alpha }{p^{n}}\right)\geq M\left(\varkappa_{0},\varkappa_{1},\frac{\alpha }{5p^{n}Q\left(\varkappa_{0},\varkappa_{1}\right)}\right)*M\left(\varkappa_{0},\varkappa_{1},\frac{\alpha }{5p^{n+1}W\left(\varkappa_{1},\varkappa_{2}\right)}\right)\\ {}*M\left(\varkappa_{0},\varkappa_{1},\frac{\alpha }{5p^{n+2}E\left(\varkappa_{2},\varkappa_{3}\right)}\right)*M\left(\varkappa_{0},\varkappa_{1},\frac{\alpha }{5p^{n+3}R\left(\varkappa_{3},\varkappa_{4}\right)}\right)*M\left(\varkappa_{0},\varkappa_{1},\frac{\alpha }{{\left(5\right)}^{2}p^{n+4}T\left(\varkappa_{4},\varkappa_{4m+1}\right)Q\left(\varkappa_{4},\varkappa_{5}\right)}\right)\\ {}*M\left(\varkappa_{0},\varkappa_{1},\frac{\alpha }{{\left(5\right)}^{2}p^{n+5}T\left(\varkappa_{4},\varkappa_{4m+1}\right)W\left(\varkappa_{5},\varkappa_{6}\right)}\right)*M\left(\varkappa_{0},\varkappa_{1},\frac{\alpha }{{\left(5\right)}^{2}p^{n+6}T\left(\varkappa_{4},\varkappa_{4m+1}\right)E\left(\varkappa_{6},\varkappa_{7}\right)}\right)\\ {}*M\left(\varkappa_{0},\varkappa_{1},\frac{\alpha }{{\left(5\right)}^{2}p^{n+7}T\left(\varkappa_{4},\varkappa_{4m+1}\right)R\left(\varkappa_{7},\varkappa_{8}\right)}\right)*M\left(\varkappa_{0},\varkappa_{1},\frac{\alpha }{{\left(5\right)}^{3}p^{n+8}T\left(\varkappa_{4},\varkappa_{4m+1}\right)T\left(\varkappa_{8},\varkappa_{4m+1}\right)Q\left(\varkappa_{8},\varkappa_{9}\right)}\right)\\ {}*M\left(\varkappa_{0},\varkappa_{1},\frac{\alpha }{{\left(5\right)}^{3}p^{n+9}T\left(\varkappa_{4},\varkappa_{4m+1}\right)T\left(\varkappa_{8},\varkappa_{4m+1}\right)W\left(\varkappa_{9},\varkappa_{10}\right)}\right)*M\left(\varkappa_{0},\varkappa_{1},\frac{\alpha }{{\left(5\right)}^{3}p^{n+10}T\left(\varkappa_{4},\varkappa_{4m+1}\right)T\left(\varkappa_{8},\varkappa_{4m+1}\right)E\left(\varkappa_{10},\varkappa_{11}\right)}\right)\\ {}*M\left(\varkappa_{0},\varkappa_{1},\frac{\alpha }{{\left(5\right)}^{3}p^{n+11}T\left(\varkappa_{4},\varkappa_{4m+1}\right)T\left(\varkappa_{8},\varkappa_{4m+1}\right)R\left(\varkappa_{11},\varkappa_{12}\right)}\right)\\ {}*M\left(\varkappa_{0},\varkappa_{1},\frac{\alpha }{{\left(5\right)}^{4}p^{n+12}T\left(\varkappa_{4},\varkappa_{4m+1}\right)T\left(\varkappa_{8},\varkappa_{4m+1}\right)T\left(\varkappa_{12},\varkappa_{4m+1}\right)Q\left(\varkappa_{12},\varkappa_{13}\right)}\right) \end{array}$$

$$\begin{array}{c} {}*\\ \vdots \\ {}*\\ M\left(\varkappa_{0},\varkappa_{1},\frac{\alpha }{{\left(5\right)}^{m}p^{n+4m}T\left(\varkappa_{4},\varkappa_{4m+1}\right)T\left(\varkappa_{8},\varkappa_{4m+1}\right)T\left(\varkappa_{12},\varkappa_{4m+1}\right)\cdots T\left(\varkappa_{4m-4},\varkappa_{4m+1}\right)T\left(\varkappa_{4m},\varkappa_{4m+1}\right)}\right) \end{array}$$ (20)

and

$$N\left(\varkappa_{n},\varkappa_{n+4m+1},\alpha \right)\leq N\left(\varkappa_{0},\varkappa_{4m+1},\frac{\alpha }{p^{n}}\right)\leq N\left(\varkappa_{0},\varkappa_{1},\frac{\alpha }{5p^{n}Q\left(\varkappa_{0},\varkappa_{1}\right)}\right)\Delta \;N\left(\varkappa_{0},\varkappa_{1},\frac{\alpha }{5p^{n+1}W\left(\varkappa_{1},\varkappa_{2}\right)}\right)\Delta \;N\left(\varkappa_{0},\varkappa_{1},\frac{\alpha }{5p^{n+2}E\left(\varkappa_{2},\varkappa_{3}\right)}\right)$$

$$\Delta \;N\left(\varkappa_{0},\varkappa_{1},\frac{\alpha }{{\left(5\right)}^{2}p^{n+4}T\left(\varkappa_{4},\varkappa_{4m+1}\right)Q\left(\varkappa_{4},\varkappa_{5}\right)}\right)\Delta \;N\left(\varkappa_{0},\varkappa_{1},\frac{\alpha }{{\left(5\right)}^{2}p^{n+5}T\left(\varkappa_{4},\varkappa_{4m+1}\right)W\left(\varkappa_{5},\varkappa_{6}\right)}\right)$$

$$\Delta \;N\left(\varkappa_{0},\varkappa_{1},\frac{\alpha }{{\left(5\right)}^{2}p^{n+6}T\left(\varkappa_{4},\varkappa_{4m+1}\right)E\left(\varkappa_{6},\varkappa_{7}\right)}\right)\Delta \;N\left(\varkappa_{0},\varkappa_{1},\frac{\alpha }{{\left(5\right)}^{2}p^{n+7}T\left(\varkappa_{4},\varkappa_{4m+1}\right)R\left(\varkappa_{7},\varkappa_{8}\right)}\right)$$

$$\Delta \;N\left(\varkappa_{0},\varkappa_{1},\frac{\alpha }{{\left(5\right)}^{3}p^{n+8}T\left(\varkappa_{4},\varkappa_{4m+1}\right)T\left(\varkappa_{8},\varkappa_{4m+1}\right)Q\left(\varkappa_{8},\varkappa_{9}\right)}\right)$$

$$\Delta \;N\left(\varkappa_{0},\varkappa_{1},\frac{\alpha }{{\left(5\right)}^{3}p^{n+9}T\left(\varkappa_{4},\varkappa_{4m+1}\right)T\left(\varkappa_{8},\varkappa_{4m+1}\right)W\left(\varkappa_{9},\varkappa_{10}\right)}\right)$$

$$\Delta \;N\left(\varkappa_{0},\varkappa_{1},\frac{\alpha }{{\left(5\right)}^{3}p^{n+10}T\left(\varkappa_{4},\varkappa_{4m+1}\right)T\left(\varkappa_{8},\varkappa_{4m+1}\right)E\left(\varkappa_{10},\varkappa_{11}\right)}\right)$$

$$\Delta \;N\left(\varkappa_{0},\varkappa_{1},\frac{\alpha }{{\left(5\right)}^{3}p^{n+11}T\left(\varkappa_{4},\varkappa_{4m+1}\right)T\left(\varkappa_{8},\varkappa_{4m+1}\right)R\left(\varkappa_{11},\varkappa_{12}\right)}\right)$$

$$\Delta \;N\left(\varkappa_{0},\varkappa_{1},\frac{\alpha }{{\left(5\right)}^{4}p^{n+12}T\left(\varkappa_{4},\varkappa_{4m+1}\right)T\left(\varkappa_{8},\varkappa_{4m+1}\right)T\left(\varkappa_{12},\varkappa_{4m+1}\right)Q\left(\varkappa_{12},\varkappa_{13}\right)}\right)$$

$$\begin{array}{c} \Delta \\ \vdots \\ \Delta \\ N\left(\varkappa_{0},\varkappa_{1},\frac{\alpha }{{\left(5\right)}^{m}p^{n+4m}T\left(\varkappa_{4},\varkappa_{4m+1}\right)T\left(\varkappa_{8},\varkappa_{4m+1}\right)T\left(\varkappa_{12},\varkappa_{4m+1}\right)\cdots T\left(\varkappa_{4m-4},\varkappa_{4m+1}\right)T\left(\varkappa_{4m},\varkappa_{4m+1}\right)}\right) \end{array}$$ (21)

Furthermore, from (4), (5), (12) and (13), we can obtain

$$\begin{array}{c} M\left(\varkappa_{0},\varkappa_{6},\alpha \right)\geq M\left(\varkappa_{0},\varkappa_{1},\frac{\alpha }{5Q\left(\varkappa_{0},\varkappa_{1}\right)}\right)*M\left(\varkappa_{0},\varkappa_{1},\frac{\alpha }{5pW\left(\varkappa_{1},\varkappa_{2}\right)}\right)\\ {}*M\left(\varkappa_{0},\varkappa_{1},\frac{\alpha }{5p^{2}E\left(\varkappa_{2},\varkappa_{3}\right)}\right)*M\left(\varkappa_{0},\varkappa_{1},\frac{\alpha }{5p^{3}R\left(\varkappa_{3},\varkappa_{4}\right)}\right)*M\left(\varkappa_{0},\varkappa_{2},\frac{\alpha }{5p^{4}T\left(\varkappa_{4},\varkappa_{6}\right)}\right), \end{array}$$

and

$$\begin{array}{c} N\left(\varkappa_{0},\varkappa_{6},\alpha \right)\leq N\left(\varkappa_{0},\varkappa_{1},\frac{\alpha }{5Q\left(\varkappa_{0},\varkappa_{1}\right)}\right)\Delta \;N\left(\varkappa_{0},\varkappa_{1},\frac{\alpha }{5pW\left(\varkappa_{1},\varkappa_{2}\right)}\right)\Delta \;N\left(\varkappa_{0},\varkappa_{1},\frac{\alpha }{5p^{2}E\left(\varkappa_{2},\varkappa_{3}\right)}\right)\\ \Delta \;N\left(\varkappa_{0},\varkappa_{1},\frac{\alpha }{5p^{3}R\left(\varkappa_{3},\varkappa_{4}\right)}\right)\Delta \;N\left(\varkappa_{0},\varkappa_{2},\frac{\alpha }{5p^{4}T\left(\varkappa_{4},\varkappa_{6}\right)}\right), \end{array}$$

In similar manner, we can deduce

$$\begin{array}{c} M\left(\varkappa_{0},\varkappa_{10},\alpha \right)\geq M\left(\varkappa_{0},\varkappa_{1},\frac{\alpha }{5Q\left(\varkappa_{0},\varkappa_{1}\right)}\right)*M\left(\varkappa_{1},\varkappa_{2},\frac{\alpha }{5W\left(\varkappa_{1},\varkappa_{2}\right)}\right)\\ {}*M\left(\varkappa_{2},\varkappa_{3},\frac{\alpha }{5E\left(\varkappa_{2},\varkappa_{3}\right)}\right)*M\left(\varkappa_{3},\varkappa_{4},\frac{\alpha }{5R\left(\varkappa_{3},\varkappa_{4}\right)}\right)*M\left(\varkappa_{4},\varkappa_{10},\frac{\alpha }{5T\left(\varkappa_{4},\varkappa_{10}\right)}\right)\\ \geq M\left(\varkappa_{0},\varkappa_{1},\frac{\alpha }{5Q\left(\varkappa_{0},\varkappa_{1}\right)}\right)*M\left(\varkappa_{0},\varkappa_{1},\frac{\alpha }{5pW\left(\varkappa_{1},\varkappa_{2}\right)}\right)*M\left(\varkappa_{0},\varkappa_{1},\frac{\alpha }{5p^{2}E\left(\varkappa_{2},\varkappa_{3}\right)}\right)\\ {}*M\left(\varkappa_{0},\varkappa_{1},\frac{\alpha }{5p^{3}R\left(\varkappa_{3},\varkappa_{4}\right)}\right)*M\left(\varkappa_{0},\varkappa_{1},\frac{\alpha }{{\left(5\right)}^{2}p^{4}T\left(\varkappa_{4},\varkappa_{9}\right)Q\left(\varkappa_{4},\varkappa_{5}\right)}\right)\\ {}*M\left(\varkappa_{0},\varkappa_{1},\frac{\alpha }{{\left(5\right)}^{2}p^{5}T\left(\varkappa_{4},\varkappa_{9}\right)W\left(\varkappa_{5},\varkappa_{6}\right)}\right)*M\left(\varkappa_{0},\varkappa_{1},\frac{\alpha }{{\left(5\right)}^{2}p^{6}T\left(\varkappa_{4},\varkappa_{9}\right)E\left(\varkappa_{6},\varkappa_{7}\right)}\right)\\ {}*M\left(\varkappa_{0},\varkappa_{1},\frac{\alpha }{{\left(5\right)}^{2}p^{7}T\left(\varkappa_{4},\varkappa_{9}\right)R\left(\varkappa_{7},\varkappa_{8}\right)}\right)*M\left(\varkappa_{0},\varkappa_{2},\frac{\alpha }{{\left(5\right)}^{2}p^{8}T\left(\varkappa_{4},\varkappa_{9}\right)T\left(\varkappa_{8},\varkappa_{10}\right)}\right), \end{array}$$

and

$$N\left(\varkappa_{0},\varkappa_{10},\alpha \right)\leq N\left(\varkappa_{0},\varkappa_{1},\frac{\alpha }{5Q\left(\varkappa_{0},\varkappa_{1}\right)}\right)\Delta \;N\left(\varkappa_{1},\varkappa_{2},\frac{\alpha }{5W\left(\varkappa_{1},\varkappa_{2}\right)}\right)\Delta \;N\left(\varkappa_{2},\varkappa_{3},\frac{\alpha }{5E\left(\varkappa_{2},\varkappa_{3}\right)}\right)$$

$$\Delta \;N\left(\varkappa_{3},\varkappa_{4},\frac{\alpha }{5R\left(\varkappa_{3},\varkappa_{4}\right)}\right)\Delta \;N\left(\varkappa_{4},\varkappa_{10},\frac{\alpha }{5T\left(\varkappa_{4},\varkappa_{10}\right)}\right)$$

$$\leq N\left(\varkappa_{0},\varkappa_{1},\frac{\alpha }{5Q\left(\varkappa_{0},\varkappa_{1}\right)}\right)\Delta \;N\left(\varkappa_{0},\varkappa_{1},\frac{\alpha }{5pW\left(\varkappa_{1},\varkappa_{2}\right)}\right)\Delta \;N\left(\varkappa_{0},\varkappa_{1},\frac{\alpha }{5p^{2}E\left(\varkappa_{2},\varkappa_{3}\right)}\right)\Delta \;N\left(\varkappa_{0},\varkappa_{1},\frac{\alpha }{5p^{3}R\left(\varkappa_{3},\varkappa_{4}\right)}\right)$$

$$\Delta \;N\left(\varkappa_{0},\varkappa_{1},\frac{\alpha }{{\left(5\right)}^{2}p^{4}T\left(\varkappa_{4},\varkappa_{9}\right)Q\left(\varkappa_{4},\varkappa_{5}\right)}\right)\Delta \;N\left(\varkappa_{0},\varkappa_{1},\frac{\alpha }{{\left(5\right)}^{2}p^{5}T\left(\varkappa_{4},\varkappa_{9}\right)W\left(\varkappa_{5},\varkappa_{6}\right)}\right)$$

$$\Delta \;N\left(\varkappa_{0},\varkappa_{1},\frac{\alpha }{{\left(5\right)}^{2}p^{6}T\left(\varkappa_{4},\varkappa_{9}\right)E\left(\varkappa_{6},\varkappa_{7}\right)}\right)\Delta \;N\left(\varkappa_{0},\varkappa_{1},\frac{\alpha }{{\left(5\right)}^{2}p^{7}T\left(\varkappa_{4},\varkappa_{9}\right)R\left(\varkappa_{7},\varkappa_{8}\right)}\right)$$

$$\Delta \;N\left(\varkappa_{0},\varkappa_{2},\frac{\alpha }{{\left(5\right)}^{2}p^{8}T\left(\varkappa_{4},\varkappa_{9}\right)T\left(\varkappa_{8},\varkappa_{10}\right)}\right).$$

We obtain for each *m* = 1,2,3,⋯,

$$\begin{array}{c} M\left(\varkappa_{0},\varkappa_{4m+2},\alpha \right)\geq M\left(\varkappa_{0},\varkappa_{1},\frac{\alpha }{5Q\left(\varkappa_{0},\varkappa_{1}\right)}\right)*M\left(\varkappa_{1},\varkappa_{2},\frac{\alpha }{5W\left(\varkappa_{1},\varkappa_{2}\right)}\right)\\ {}*M\left(\varkappa_{2},\varkappa_{3},\frac{\alpha }{5E\left(\varkappa_{2},\varkappa_{3}\right)}\right)*M\left(\varkappa_{3},\varkappa_{4},\frac{\alpha }{5R\left(\varkappa_{3},\varkappa_{4}\right)}\right)*M\left(\varkappa_{4},\varkappa_{4m+2},\frac{\alpha }{5T\left(\varkappa_{4},\varkappa_{4m+2}\right)}\right) \end{array}$$

$$\begin{array}{c} \geq M\left(\varkappa_{0},\varkappa_{1},\frac{\alpha }{5Q\left(\varkappa_{0},\varkappa_{1}\right)}\right)*M\left(\varkappa_{0},\varkappa_{1},\frac{\alpha }{5pW\left(\varkappa_{1},\varkappa_{2}\right)}\right)*M\left(\varkappa_{0},\varkappa_{1},\frac{\alpha }{5p^{2}E\left(\varkappa_{2},\varkappa_{3}\right)}\right)*M\left(\varkappa_{0},\varkappa_{1},\frac{\alpha }{5p^{3}R\left(\varkappa_{3},\varkappa_{4}\right)}\right)\\ {}*M\left(\varkappa_{0},\varkappa_{1},\frac{\alpha }{{\left(5\right)}^{2}p^{4}T\left(\varkappa_{4},\varkappa_{4m+2}\right)Q\left(\varkappa_{4},\varkappa_{5}\right)}\right)*M\left(\varkappa_{0},\varkappa_{1},\frac{\alpha }{{\left(5\right)}^{2}p^{5}T\left(\varkappa_{4},\varkappa_{4m+2}\right)W\left(\varkappa_{5},\varkappa_{6}\right)}\right)\\ {}*M\left(\varkappa_{0},\varkappa_{1},\frac{\alpha }{{\left(5\right)}^{2}p^{6}T\left(\varkappa_{4},\varkappa_{4m+2}\right)E\left(\varkappa_{6},\varkappa_{7}\right)}\right)*M\left(\varkappa_{0},\varkappa_{1},\frac{\alpha }{{\left(5\right)}^{2}p^{7}T\left(\varkappa_{4},\varkappa_{4m+2}\right)R\left(\varkappa_{7},\varkappa_{8}\right)}\right)\\ {}*M\left(\varkappa_{0},\varkappa_{1},\frac{\alpha }{{\left(5\right)}^{3}p^{8}T\left(\varkappa_{4},\varkappa_{4m+2}\right)T\left(\varkappa_{8},\varkappa_{4m+2}\right)Q\left(\varkappa_{8},\varkappa_{9}\right)}\right)*M\left(\varkappa_{0},\varkappa_{1},\frac{\alpha }{{\left(5\right)}^{3}p^{9}T\left(\varkappa_{4},\varkappa_{4m+2}\right)T\left(\varkappa_{8},\varkappa_{4m+2}\right)W\left(\varkappa_{9},\varkappa_{10}\right)}\right)\\ {}*M\left(\varkappa_{0},\varkappa_{1},\frac{\alpha }{{\left(5\right)}^{3}p^{10}T\left(\varkappa_{4},\varkappa_{4m+2}\right)T\left(\varkappa_{8},\varkappa_{4m+2}\right)E\left(\varkappa_{10},\varkappa_{11}\right)}\right)\\ {}*M\left(\varkappa_{0},\varkappa_{1},\frac{\alpha }{{\left(5\right)}^{3}p^{11}T\left(\varkappa_{4},\varkappa_{4m+2}\right)T\left(\varkappa_{8},\varkappa_{4m+2}\right)R\left(\varkappa_{11},\varkappa_{12}\right)}\right)\\ {}*M\left(\varkappa_{0},\varkappa_{1},\frac{\alpha }{{\left(5\right)}^{4}p^{12}T\left(\varkappa_{4},\varkappa_{4m+2}\right)T\left(\varkappa_{8},\varkappa_{4m+2}\right)T\left(\varkappa_{12},\varkappa_{4m+2}\right)Q\left(\varkappa_{12},\varkappa_{13}\right)}\right) \end{array}$$

$$\begin{array}{c} {}*\\ \vdots \\ {}*\\ M\left(\varkappa_{0},\varkappa_{2},\frac{\alpha }{{\left(5\right)}^{m}p^{4m}T\left(\varkappa_{4},\varkappa_{4m+2}\right)T\left(\varkappa_{8},\varkappa_{4m+2}\right)T\left(\varkappa_{12},\varkappa_{4m+2}\right)\cdots T\left(\varkappa_{4m-4},\varkappa_{4m+2}\right)T\left(\varkappa_{4m},\varkappa_{4m+2}\right)}\right) \end{array}$$

and

$$N\left(\varkappa_{0},\varkappa_{4m+2},\alpha \right)\leq N\left(\varkappa_{0},\varkappa_{1},\frac{\alpha }{5Q\left(\varkappa_{0},\varkappa_{1}\right)}\right)\Delta \;N\left(\varkappa_{1},\varkappa_{2},\frac{\alpha }{5W\left(\varkappa_{1},\varkappa_{2}\right)}\right)\Delta \;N\left(\varkappa_{2},\varkappa_{3},\frac{\alpha }{5E\left(\varkappa_{2},\varkappa_{3}\right)}\right)$$

$$\Delta \;N\left(\varkappa_{3},\varkappa_{4},\frac{\alpha }{5R\left(\varkappa_{3},\varkappa_{4}\right)}\right)\Delta \;N\left(\varkappa_{4},\varkappa_{4m+2},\frac{\alpha }{5T\left(\varkappa_{4},\varkappa_{4m+2}\right)}\right)$$

$$\leq N\left(\varkappa_{0},\varkappa_{1},\frac{\alpha }{5Q\left(\varkappa_{0},\varkappa_{1}\right)}\right)\Delta \;N\left(\varkappa_{0},\varkappa_{1},\frac{\alpha }{5pW\left(\varkappa_{1},\varkappa_{2}\right)}\right)\Delta \;N\left(\varkappa_{0},\varkappa_{1},\frac{\alpha }{5p^{2}E\left(\varkappa_{2},\varkappa_{3}\right)}\right)\Delta \;N\left(\varkappa_{0},\varkappa_{1},\frac{\alpha }{5p^{3}R\left(\varkappa_{3},\varkappa_{4}\right)}\right)$$

$$\Delta \;N\left(\varkappa_{0},\varkappa_{1},\frac{\alpha }{{\left(5\right)}^{2}p^{4}T\left(\varkappa_{4},\varkappa_{4m+2}\right)Q\left(\varkappa_{4},\varkappa_{5}\right)}\right)\Delta \;N\left(\varkappa_{0},\varkappa_{1},\frac{\alpha }{{\left(5\right)}^{2}p^{5}T\left(\varkappa_{4},\varkappa_{4m+2}\right)W\left(\varkappa_{5},\varkappa_{6}\right)}\right)$$

$$\Delta \;N\left(\varkappa_{0},\varkappa_{1},\frac{\alpha }{{\left(5\right)}^{2}p^{6}T\left(\varkappa_{4},\varkappa_{4m+2}\right)E\left(\varkappa_{6},\varkappa_{7}\right)}\right)\Delta \;N\left(\varkappa_{0},\varkappa_{1},\frac{\alpha }{{\left(5\right)}^{2}p^{7}T\left(\varkappa_{4},\varkappa_{4m+2}\right)R\left(\varkappa_{7},\varkappa_{8}\right)}\right)$$

$$\Delta \;N\left(\varkappa_{0},\varkappa_{1},\frac{\alpha }{{\left(5\right)}^{3}p^{8}T\left(\varkappa_{4},\varkappa_{4m+2}\right)T\left(\varkappa_{8},\varkappa_{4m+2}\right)Q\left(\varkappa_{8},\varkappa_{9}\right)}\right)$$

$$\Delta \;N\left(\varkappa_{0},\varkappa_{1},\frac{\alpha }{{\left(5\right)}^{3}p^{9}T\left(\varkappa_{4},\varkappa_{4m+2}\right)T\left(\varkappa_{8},\varkappa_{4m+2}\right)W\left(\varkappa_{9},\varkappa_{10}\right)}\right)$$

$$\Delta \;N\left(\varkappa_{0},\varkappa_{1},\frac{\alpha }{{\left(5\right)}^{3}p^{10}T\left(\varkappa_{4},\varkappa_{4m+2}\right)T\left(\varkappa_{8},\varkappa_{4m+2}\right)E\left(\varkappa_{10},\varkappa_{11}\right)}\right)$$

$$\Delta \;N\left(\varkappa_{0},\varkappa_{1},\frac{\alpha }{{\left(5\right)}^{3}p^{11}T\left(\varkappa_{4},\varkappa_{4m+2}\right)T\left(\varkappa_{8},\varkappa_{4m+2}\right)R\left(\varkappa_{11},\varkappa_{12}\right)}\right)$$

$$\Delta \;N\left(\varkappa_{0},\varkappa_{1},\frac{\alpha }{{\left(5\right)}^{4}p^{12}T\left(\varkappa_{4},\varkappa_{4m+2}\right)T\left(\varkappa_{8},\varkappa_{4m+2}\right)T\left(\varkappa_{12},\varkappa_{4m+2}\right)Q\left(\varkappa_{12},\varkappa_{13}\right)}\right)$$

$$\begin{array}{c} \Delta \\ \vdots \\ \Delta \\ N\left(\varkappa_{0},\varkappa_{2},\frac{\alpha }{{\left(5\right)}^{m}p^{4m}T\left(\varkappa_{4},\varkappa_{4m+2}\right)T\left(\varkappa_{8},\varkappa_{4m+2}\right)T\left(\varkappa_{12},\varkappa_{4m+2}\right)\cdots T\left(\varkappa_{4m-4},\varkappa_{4m+2}\right)T\left(\varkappa_{4m},\varkappa_{4m+2}\right)}\right). \end{array}$$

Now, using (9) and (17), we deduce that

$$\begin{array}{c} M\left(\varkappa_{n},\varkappa_{n+4m+2},\alpha \right)\geq M\left(\varkappa_{0},\varkappa_{4m+2},\frac{\alpha }{p^{n}}\right)\;\geq M\left(\varkappa_{0},\varkappa_{1},\frac{\alpha }{5p^{n}Q\left(\varkappa_{0},\varkappa_{1}\right)}\right)*M\left(\varkappa_{0},\varkappa_{1},\frac{\alpha }{5p^{n+1}W\left(\varkappa_{1},\varkappa_{2}\right)}\right)\\ {}*M\left(\varkappa_{0},\varkappa_{1},\frac{\alpha }{5p^{n+2}E\left(\varkappa_{2},\varkappa_{3}\right)}\right)*M\left(\varkappa_{0},\varkappa_{1},\frac{\alpha }{5p^{n+3}R\left(\varkappa_{3},\varkappa_{4}\right)}\right)\\ {}*M\left(\varkappa_{0},\varkappa_{1},\frac{\alpha }{{\left(5\right)}^{2}p^{n+4}T\left(\varkappa_{4},\varkappa_{4m+2}\right)Q\left(\varkappa_{4},\varkappa_{5}\right)}\right)\\ {}*M\left(\varkappa_{0},\varkappa_{1},\frac{\alpha }{{\left(5\right)}^{2}p^{n+5}T\left(\varkappa_{4},\varkappa_{4m+2}\right)W\left(\varkappa_{5},\varkappa_{6}\right)}\right)*M\left(\varkappa_{0},\varkappa_{1},\frac{\alpha }{{\left(5\right)}^{2}p^{n+6}T\left(\varkappa_{4},\varkappa_{4m+2}\right)E\left(\varkappa_{6},\varkappa_{7}\right)}\right)\\ {}*M\left(\varkappa_{0},\varkappa_{1},\frac{\alpha }{{\left(5\right)}^{2}p^{n+7}T\left(\varkappa_{4},\varkappa_{4m+2}\right)R\left(\varkappa_{7},\varkappa_{8}\right)}\right)*M\left(\varkappa_{0},\varkappa_{1},\frac{\alpha }{{\left(5\right)}^{3}p^{n+8}T\left(\varkappa_{4},\varkappa_{4m+2}\right)T\left(\varkappa_{8},\varkappa_{4m+2}\right)Q\left(\varkappa_{8},\varkappa_{9}\right)}\right)\\ {}*M\left(\varkappa_{0},\varkappa_{1},\frac{\alpha }{{\left(5\right)}^{3}p^{n+9}T\left(\varkappa_{4},\varkappa_{4m+2}\right)T\left(\varkappa_{8},\varkappa_{4m+2}\right)W\left(\varkappa_{9},\varkappa_{10}\right)}\right)\\ {}*M\left(\varkappa_{0},\varkappa_{1},\frac{\alpha }{{\left(5\right)}^{3}p^{n+10}T\left(\varkappa_{4},\varkappa_{4m+2}\right)T\left(\varkappa_{8},\varkappa_{4m+2}\right)E\left(\varkappa_{10},\varkappa_{11}\right)}\right)\\ {}*M\left(\varkappa_{0},\varkappa_{1},\frac{\alpha }{{\left(5\right)}^{3}p^{n+11}T\left(\varkappa_{4},\varkappa_{4m+2}\right)T\left(\varkappa_{8},\varkappa_{4m+2}\right)R\left(\varkappa_{11},\varkappa_{12}\right)}\right)\\ {}*M\left(\varkappa_{0},\varkappa_{1},\frac{\alpha }{{\left(5\right)}^{4}p^{n+12}T\left(\varkappa_{4},\varkappa_{4m+2}\right)T\left(\varkappa_{8},\varkappa_{4m+2}\right)T\left(\varkappa_{12},\varkappa_{4m+2}\right)Q\left(\varkappa_{12},\varkappa_{13}\right)}\right) \end{array}$$

$$\begin{array}{c} {}*\\ \vdots \\ {}*\\ M\left(\varkappa_{0},\varkappa_{2},\frac{\alpha }{{\left(5\right)}^{m}p^{n+4m}T\left(\varkappa_{4},\varkappa_{4m+2}\right)T\left(\varkappa_{8},\varkappa_{4m+2}\right)T\left(\varkappa_{12},\varkappa_{4m+2}\right)\cdots T\left(\varkappa_{4m-4},\varkappa_{4m+2}\right)T\left(\varkappa_{4m},\varkappa_{4m+2}\right)}\right) \end{array}$$ (22)

and

$$N\left(\varkappa_{n},\varkappa_{n+4m+2},\alpha \right)\leq N\left(\varkappa_{0},\varkappa_{4m+2},\frac{\alpha }{p^{n}}\right)\leq N\left(\varkappa_{0},\varkappa_{1},\frac{\alpha }{5p^{n}Q\left(\varkappa_{0},\varkappa_{1}\right)}\right)\Delta \;N\left(\varkappa_{0},\varkappa_{1},\frac{\alpha }{5p^{n+1}W\left(\varkappa_{1},\varkappa_{2}\right)}\right)\Delta \;N\left(\varkappa_{0},\varkappa_{1},\frac{\alpha }{5p^{n+2}E\left(\varkappa_{2},\varkappa_{3}\right)}\right)$$

$$\Delta \;N\left(\varkappa_{0},\varkappa_{1},\frac{\alpha }{5p^{n+3}R\left(\varkappa_{3},\varkappa_{4}\right)}\right)\Delta \;N\left(\varkappa_{0},\varkappa_{1},\frac{\alpha }{{\left(5\right)}^{2}p^{n+4}T\left(\varkappa_{4},\varkappa_{4m+2}\right)Q\left(\varkappa_{4},\varkappa_{5}\right)}\right)$$

$$\Delta \;N\left(\varkappa_{0},\varkappa_{1},\frac{\alpha }{{\left(5\right)}^{2}p^{n+5}T\left(\varkappa_{4},\varkappa_{4m+2}\right)W\left(\varkappa_{5},\varkappa_{6}\right)}\right)\Delta \;N\left(\varkappa_{0},\varkappa_{1},\frac{\alpha }{{\left(5\right)}^{2}p^{n+6}T\left(\varkappa_{4},\varkappa_{4m+2}\right)E\left(\varkappa_{6},\varkappa_{7}\right)}\right)$$

$$\Delta \;N\left(\varkappa_{0},\varkappa_{1},\frac{\alpha }{{\left(5\right)}^{2}p^{n+7}T\left(\varkappa_{4},\varkappa_{4m+2}\right)R\left(\varkappa_{7},\varkappa_{8}\right)}\right)\Delta \;N\left(\varkappa_{0},\varkappa_{1},\frac{\alpha }{{\left(5\right)}^{3}p^{n+8}T\left(\varkappa_{4},\varkappa_{4m+2}\right)T\left(\varkappa_{8},\varkappa_{4m+2}\right)Q\left(\varkappa_{8},\varkappa_{9}\right)}\right)$$

$$\Delta \;N\left(\varkappa_{0},\varkappa_{1},\frac{\alpha }{{\left(5\right)}^{3}p^{n+9}T\left(\varkappa_{4},\varkappa_{4m+2}\right)T\left(\varkappa_{8},\varkappa_{4m+2}\right)W\left(\varkappa_{9},\varkappa_{10}\right)}\right)$$

$$\Delta \;N\left(\varkappa_{0},\varkappa_{1},\frac{\alpha }{{\left(5\right)}^{3}p^{n+10}T\left(\varkappa_{4},\varkappa_{4m+2}\right)T\left(\varkappa_{8},\varkappa_{4m+2}\right)E\left(\varkappa_{10},\varkappa_{11}\right)}\right)$$

$$\Delta \;N\left(\varkappa_{0},\varkappa_{1},\frac{\alpha }{{\left(5\right)}^{3}p^{n+11}T\left(\varkappa_{4},\varkappa_{4m+2}\right)T\left(\varkappa_{8},\varkappa_{4m+2}\right)R\left(\varkappa_{11},\varkappa_{12}\right)}\right)$$

$$\Delta \;N\left(\varkappa_{0},\varkappa_{1},\frac{\alpha }{{\left(5\right)}^{4}p^{n+12}T\left(\varkappa_{4},\varkappa_{4m+2}\right)T\left(\varkappa_{8},\varkappa_{4m+2}\right)T\left(\varkappa_{12},\varkappa_{4m+2}\right)Q\left(\varkappa_{12},\varkappa_{13}\right)}\right)$$

$$\begin{array}{c} \Delta \\ \vdots \\ \Delta \\ N\left(\varkappa_{0},\varkappa_{2},\frac{\alpha }{{\left(5\right)}^{m}p^{n+4m}T\left(\varkappa_{4},\varkappa_{4m+2}\right)T\left(\varkappa_{8},\varkappa_{4m+2}\right)T\left(\varkappa_{12},\varkappa_{4m+2}\right)\cdots T\left(\varkappa_{4m-4},\varkappa_{4m+2}\right)T\left(\varkappa_{4m},\varkappa_{4m+2}\right)}\right), \end{array}$$ (23)

Accordingly, we get

$$\begin{array}{c} M\left(\varkappa_{n},\varkappa_{n+4m+3},\alpha \right)\geq M\left(\varkappa_{0},\varkappa_{4m+3},\frac{\alpha }{p^{n}}\right)\;\geq M\left(\varkappa_{0},\varkappa_{1},\frac{\alpha }{5p^{n}Q\left(\varkappa_{0},\varkappa_{1}\right)}\right)\\ {}*M\left(\varkappa_{0},\varkappa_{1},\frac{\alpha }{5p^{n+1}W\left(\varkappa_{1},\varkappa_{2}\right)}\right)*M\left(\varkappa_{0},\varkappa_{1},\frac{\alpha }{5p^{n+2}E\left(\varkappa_{2},\varkappa_{3}\right)}\right)*M\left(\varkappa_{0},\varkappa_{1},\frac{\alpha }{5p^{n+3}R\left(\varkappa_{3},\varkappa_{4}\right)}\right)\\ {}*M\left(\varkappa_{0},\varkappa_{1},\frac{\alpha }{{\left(5\right)}^{2}p^{n+4}T\left(\varkappa_{4},\varkappa_{4m+3}\right)Q\left(\varkappa_{4},\varkappa_{5}\right)}\right)*M\left(\varkappa_{0},\varkappa_{1},\frac{\alpha }{{\left(5\right)}^{2}p^{n+5}T\left(\varkappa_{4},\varkappa_{4m+3}\right)W\left(\varkappa_{5},\varkappa_{6}\right)}\right)\\ {}*M\left(\varkappa_{0},\varkappa_{1},\frac{\alpha }{{\left(5\right)}^{2}p^{n+6}T\left(\varkappa_{4},\varkappa_{4m+3}\right)E\left(\varkappa_{6},\varkappa_{7}\right)}\right)*M\left(\varkappa_{0},\varkappa_{1},\frac{\alpha }{{\left(5\right)}^{2}p^{n+7}T\left(\varkappa_{4},\varkappa_{4m+3}\right)R\left(\varkappa_{7},\varkappa_{8}\right)}\right)\\ {}*M\left(\varkappa_{0},\varkappa_{1},\frac{\alpha }{{\left(5\right)}^{3}p^{n+8}T\left(\varkappa_{4},\varkappa_{4m+3}\right)T\left(\varkappa_{8},\varkappa_{4m+3}\right)Q\left(\varkappa_{8},\varkappa_{9}\right)}\right)\\ {}*M\left(\varkappa_{0},\varkappa_{1},\frac{\alpha }{{\left(5\right)}^{3}p^{n+9}T\left(\varkappa_{4},\varkappa_{4m+3}\right)T\left(\varkappa_{8},\varkappa_{4m+3}\right)W\left(\varkappa_{9},\varkappa_{10}\right)}\right)\\ {}*M\left(\varkappa_{0},\varkappa_{1},\frac{\alpha }{{\left(5\right)}^{3}p^{n+10}T\left(\varkappa_{4},\varkappa_{4m+3}\right)T\left(\varkappa_{8},\varkappa_{4m+3}\right)E\left(\varkappa_{10},\varkappa_{11}\right)}\right)\\ {}*M\left(\varkappa_{0},\varkappa_{1},\frac{\alpha }{{\left(5\right)}^{3}p^{n+11}T\left(\varkappa_{4},\varkappa_{4m+3}\right)T\left(\varkappa_{8},\varkappa_{4m+3}\right)R\left(\varkappa_{11},\varkappa_{12}\right)}\right)\\ {}*M\left(\varkappa_{0},\varkappa_{1},\frac{\alpha }{{\left(5\right)}^{4}p^{n+12}T\left(\varkappa_{4},\varkappa_{4m+3}\right)T\left(\varkappa_{8},\varkappa_{4m+3}\right)T\left(\varkappa_{12},\varkappa_{4m+3}\right)Q\left(\varkappa_{12},\varkappa_{13}\right)}\right) \end{array}$$

$$\begin{array}{c} {}*\\ \vdots \\ {}*\\ M\left(\varkappa_{0},\varkappa_{3},\frac{\alpha }{{\left(5\right)}^{m}p^{n+4m}T\left(\varkappa_{4},\varkappa_{4m+3}\right)T\left(\varkappa_{8},\varkappa_{4m+3}\right)T\left(\varkappa_{12},\varkappa_{4m+3}\right)\cdots T\left(\varkappa_{4m-4},\varkappa_{4m+3}\right)T\left(\varkappa_{4m},\varkappa_{4m+3}\right)}\right), \end{array}$$ (24)

$$\begin{array}{c} M\left(\varkappa_{n},\varkappa_{n+4m+4},\alpha \right)\geq M\left(\varkappa_{0},\varkappa_{4m+4},\frac{\alpha }{p^{n}}\right)\;\geq M\left(\varkappa_{0},\varkappa_{1},\frac{\alpha }{5p^{n}Q\left(\varkappa_{0},\varkappa_{1}\right)}\right)\\ {}*M\left(\varkappa_{0},\varkappa_{1},\frac{\alpha }{5p^{n+1}W\left(\varkappa_{1},\varkappa_{2}\right)}\right)*M\left(\varkappa_{0},\varkappa_{1},\frac{\alpha }{5p^{n+2}E\left(\varkappa_{2},\varkappa_{3}\right)}\right)*M\left(\varkappa_{0},\varkappa_{1},\frac{\alpha }{5p^{n+3}R\left(\varkappa_{3},\varkappa_{4}\right)}\right)\\ {}*M\left(\varkappa_{0},\varkappa_{1},\frac{\alpha }{{\left(5\right)}^{2}p^{n+4}T\left(\varkappa_{4},\varkappa_{4m+4}\right)Q\left(\varkappa_{4},\varkappa_{5}\right)}\right)*M\left(\varkappa_{0},\varkappa_{1},\frac{\alpha }{{\left(5\right)}^{2}p^{n+5}T\left(\varkappa_{4},\varkappa_{4m+4}\right)W\left(\varkappa_{5},\varkappa_{6}\right)}\right)\\ {}*M\left(\varkappa_{0},\varkappa_{1},\frac{\alpha }{{\left(5\right)}^{2}p^{n+6}T\left(\varkappa_{4},\varkappa_{4m+4}\right)E\left(\varkappa_{6},\varkappa_{7}\right)}\right)*M\left(\varkappa_{0},\varkappa_{1},\frac{\alpha }{{\left(5\right)}^{2}p^{n+7}T\left(\varkappa_{4},\varkappa_{4m+4}\right)R\left(\varkappa_{7},\varkappa_{8}\right)}\right)\\ {}*M\left(\varkappa_{0},\varkappa_{1},\frac{\alpha }{{\left(5\right)}^{3}p^{n+8}T\left(\varkappa_{4},\varkappa_{4m+4}\right)T\left(\varkappa_{8},\varkappa_{4m+4}\right)Q\left(\varkappa_{8},\varkappa_{9}\right)}\right)\\ {}*M\left(\varkappa_{0},\varkappa_{1},\frac{\alpha }{{\left(5\right)}^{3}p^{n+9}T\left(\varkappa_{4},\varkappa_{4m+4}\right)T\left(\varkappa_{8},\varkappa_{4m+4}\right)W\left(\varkappa_{9},\varkappa_{10}\right)}\right)\\ {}*M\left(\varkappa_{0},\varkappa_{1},\frac{\alpha }{{\left(5\right)}^{3}p^{n+10}T\left(\varkappa_{4},\varkappa_{4m+4}\right)T\left(\varkappa_{8},\varkappa_{4m+4}\right)E\left(\varkappa_{10},\varkappa_{11}\right)}\right)\\ {}*M\left(\varkappa_{0},\varkappa_{1},\frac{\alpha }{{\left(5\right)}^{3}p^{n+11}T\left(\varkappa_{4},\varkappa_{4m+4}\right)T\left(\varkappa_{8},\varkappa_{4m+4}\right)R\left(\varkappa_{11},\varkappa_{12}\right)}\right)\\ {}*M\left(\varkappa_{0},\varkappa_{1},\frac{\alpha }{{\left(5\right)}^{4}p^{n+12}T\left(\varkappa_{4},\varkappa_{4m+4}\right)T\left(\varkappa_{8},\varkappa_{4m+4}\right)T\left(\varkappa_{12},\varkappa_{4m+4}\right)Q\left(\varkappa_{12},\varkappa_{13}\right)}\right) \end{array}$$

$$\begin{array}{c} {}*\\ \vdots \\ {}*\\ M\left(\varkappa_{0},\varkappa_{4},\frac{\alpha }{{\left(5\right)}^{m}p^{n+4m}T\left(\varkappa_{4},\varkappa_{4m+4}\right)T\left(\varkappa_{8},\varkappa_{4m+4}\right)T\left(\varkappa_{12},\varkappa_{4m+4}\right)\cdots T\left(\varkappa_{4m-4},\varkappa_{4m+4}\right)T\left(\varkappa_{4m},\varkappa_{4m+4}\right)}\right) \end{array}$$ (25)

and

$$N\left(\varkappa_{n},\varkappa_{n+4m+3},\alpha \right)\leq N\left(\varkappa_{0},\varkappa_{4m+3},\frac{\alpha }{p^{n}}\right)\leq N\left(\varkappa_{0},\varkappa_{1},\frac{\alpha }{5p^{n}Q\left(\varkappa_{0},\varkappa_{1}\right)}\right)\Delta \;N\left(\varkappa_{0},\varkappa_{1},\frac{\alpha }{5p^{n+1}W\left(\varkappa_{1},\varkappa_{2}\right)}\right)\Delta \;N\left(\varkappa_{0},\varkappa_{1},\frac{\alpha }{5p^{n+2}E\left(\varkappa_{2},\varkappa_{3}\right)}\right)$$

$$\Delta \;N\left(\varkappa_{0},\varkappa_{1},\frac{\alpha }{5p^{n+3}R\left(\varkappa_{3},\varkappa_{4}\right)}\right)\Delta \;N\left(\varkappa_{0},\varkappa_{1},\frac{\alpha }{{\left(5\right)}^{2}p^{n+4}T\left(\varkappa_{4},\varkappa_{4m+3}\right)Q\left(\varkappa_{4},\varkappa_{5}\right)}\right)$$

$$\Delta \;N\left(\varkappa_{0},\varkappa_{1},\frac{\alpha }{{\left(5\right)}^{2}p^{n+5}T\left(\varkappa_{4},\varkappa_{4m+3}\right)W\left(\varkappa_{5},\varkappa_{6}\right)}\right)\Delta \;N\left(\varkappa_{0},\varkappa_{1},\frac{\alpha }{{\left(5\right)}^{2}p^{n+6}T\left(\varkappa_{4},\varkappa_{4m+3}\right)E\left(\varkappa_{6},\varkappa_{7}\right)}\right)$$

$$\Delta \;N\left(\varkappa_{0},\varkappa_{1},\frac{\alpha }{{\left(5\right)}^{2}p^{n+7}T\left(\varkappa_{4},\varkappa_{4m+3}\right)R\left(\varkappa_{7},\varkappa_{8}\right)}\right)\Delta \;N\left(\varkappa_{0},\varkappa_{1},\frac{\alpha }{{\left(5\right)}^{3}p^{n+8}T\left(\varkappa_{4},\varkappa_{4m+3}\right)T\left(\varkappa_{8},\varkappa_{4m+3}\right)Q\left(\varkappa_{8},\varkappa_{9}\right)}\right)$$

$$\Delta \;N\left(\varkappa_{0},\varkappa_{1},\frac{\alpha }{{\left(5\right)}^{3}p^{n+9}T\left(\varkappa_{4},\varkappa_{4m+3}\right)T\left(\varkappa_{8},\varkappa_{4m+3}\right)W\left(\varkappa_{9},\varkappa_{10}\right)}\right)$$

$$\Delta \;N\left(\varkappa_{0},\varkappa_{1},\frac{\alpha }{{\left(5\right)}^{3}p^{n+10}T\left(\varkappa_{4},\varkappa_{4m+3}\right)T\left(\varkappa_{8},\varkappa_{4m+3}\right)E\left(\varkappa_{10},\varkappa_{11}\right)}\right)$$

$$\Delta \;N\left(\varkappa_{0},\varkappa_{1},\frac{\alpha }{{\left(5\right)}^{3}p^{n+11}T\left(\varkappa_{4},\varkappa_{4m+3}\right)T\left(\varkappa_{8},\varkappa_{4m+3}\right)R\left(\varkappa_{11},\varkappa_{12}\right)}\right)$$

$$\Delta \;N\left(\varkappa_{0},\varkappa_{1},\frac{\alpha }{{\left(5\right)}^{4}p^{n+12}T\left(\varkappa_{4},\varkappa_{4m+3}\right)T\left(\varkappa_{8},\varkappa_{4m+3}\right)T\left(\varkappa_{12},\varkappa_{4m+3}\right)Q\left(\varkappa_{12},\varkappa_{13}\right)}\right)$$

$$\begin{array}{c} \Delta \\ \vdots \\ \Delta \\ N\left(\varkappa_{0},\varkappa_{3},\frac{\alpha }{{\left(5\right)}^{m}p^{n+4m}T\left(\varkappa_{4},\varkappa_{4m+3}\right)T\left(\varkappa_{8},\varkappa_{4m+3}\right)T\left(\varkappa_{12},\varkappa_{4m+3}\right)\cdots T\left(\varkappa_{4m-4},\varkappa_{4m+3}\right)T\left(\varkappa_{4m},\varkappa_{4m+3}\right)}\right), \end{array}$$ (26)

$$N\left(\varkappa_{n},\varkappa_{n+4m+4},\alpha \right)\leq N\left(\varkappa_{0},\varkappa_{4m+4},\frac{\alpha }{p^{n}}\right)\leq N\left(\varkappa_{0},\varkappa_{1},\frac{\alpha }{5p^{n}Q\left(\varkappa_{0},\varkappa_{1}\right)}\right)\Delta \;N\left(\varkappa_{0},\varkappa_{1},\frac{\alpha }{5p^{n+1}W\left(\varkappa_{1},\varkappa_{2}\right)}\right)\Delta \;N\left(\varkappa_{0},\varkappa_{1},\frac{\alpha }{5p^{n+2}E\left(\varkappa_{2},\varkappa_{3}\right)}\right)$$

$$\Delta \;N\left(\varkappa_{0},\varkappa_{1},\frac{\alpha }{5p^{n+3}R\left(\varkappa_{3},\varkappa_{4}\right)}\right)\Delta \;N\left(\varkappa_{0},\varkappa_{1},\frac{\alpha }{{\left(5\right)}^{2}p^{n+4}T\left(\varkappa_{4},\varkappa_{4m+4}\right)Q\left(\varkappa_{4},\varkappa_{5}\right)}\right)$$

$$\Delta \;N\left(\varkappa_{0},\varkappa_{1},\frac{\alpha }{{\left(5\right)}^{2}p^{n+5}T\left(\varkappa_{4},\varkappa_{4m+4}\right)W\left(\varkappa_{5},\varkappa_{6}\right)}\right)\Delta \;N\left(\varkappa_{0},\varkappa_{1},\frac{\alpha }{{\left(5\right)}^{2}p^{n+6}T\left(\varkappa_{4},\varkappa_{4m+4}\right)E\left(\varkappa_{6},\varkappa_{7}\right)}\right)$$

$$\Delta \;N\left(\varkappa_{0},\varkappa_{1},\frac{\alpha }{{\left(5\right)}^{2}p^{n+7}T\left(\varkappa_{4},\varkappa_{4m+4}\right)R\left(\varkappa_{7},\varkappa_{8}\right)}\right)\Delta \;N\left(\varkappa_{0},\varkappa_{1},\frac{\alpha }{{\left(5\right)}^{3}p^{n+8}T\left(\varkappa_{4},\varkappa_{4m+4}\right)T\left(\varkappa_{8},\varkappa_{4m+4}\right)Q\left(\varkappa_{8},\varkappa_{9}\right)}\right)$$

$$\Delta \;N\left(\varkappa_{0},\varkappa_{1},\frac{\alpha }{{\left(5\right)}^{3}p^{n+9}T\left(\varkappa_{4},\varkappa_{4m+4}\right)T\left(\varkappa_{8},\varkappa_{4m+4}\right)W\left(\varkappa_{9},\varkappa_{10}\right)}\right)$$

$$\Delta \;N\left(\varkappa_{0},\varkappa_{1},\frac{\alpha }{{\left(5\right)}^{3}p^{n+10}T\left(\varkappa_{4},\varkappa_{4m+4}\right)T\left(\varkappa_{8},\varkappa_{4m+4}\right)E\left(\varkappa_{10},\varkappa_{11}\right)}\right)$$

$$\Delta \;N\left(\varkappa_{0},\varkappa_{1},\frac{\alpha }{{\left(5\right)}^{3}p^{n+11}T\left(\varkappa_{4},\varkappa_{4m+4}\right)T\left(\varkappa_{8},\varkappa_{4m+4}\right)R\left(\varkappa_{11},\varkappa_{12}\right)}\right)$$

$$\Delta \;N\left(\varkappa_{0},\varkappa_{1},\frac{\alpha }{{\left(5\right)}^{4}p^{n+12}T\left(\varkappa_{4},\varkappa_{4m+4}\right)T\left(\varkappa_{8},\varkappa_{4m+4}\right)T\left(\varkappa_{12},\varkappa_{4m+4}\right)Q\left(\varkappa_{12},\varkappa_{13}\right)}\right)$$

$$\begin{array}{c} \Delta \\ \vdots \\ \Delta \\ N\left(\varkappa_{0},\varkappa_{4},\frac{\alpha }{{\left(5\right)}^{m}p^{n+4m}T\left(\varkappa_{4},\varkappa_{4m+4}\right)T\left(\varkappa_{8},\varkappa_{4m+4}\right)T\left(\varkappa_{12},\varkappa_{4m+4}\right)\cdots T\left(\varkappa_{4m-4},\varkappa_{4m+4}\right)T\left(\varkappa_{4m},\varkappa_{4m+4}\right)}\right). \end{array}$$ (27)

Furthermore, for every *q* and from the inequalities (20)–(27), we have

$$\underset{n\to \infty }{\lim}M\left(\varkappa_{n},\varkappa_{n+q},\alpha \right)=1*1*1*\cdots *1=1,$$ (28)

$$\underset{n\to \infty }{\lim}N\left(\varkappa_{n},\varkappa_{n+q},\alpha \right)=0\;\Delta \;0\;\Delta \;0\;\Delta \cdots \Delta \;0=0,$$ (29)

as $b\left(\varkappa_{n},\varkappa_{n+q}\right)<\frac{1}{p}\;for\;all\;n,q\in N\;and\;p\in \left(0,1\right)\;i.e.,\;\varkappa_{n}$ is a Cauchy sequence in *X*. Since, (*X*, *M*, *N*, *, Δ) is complete, there exists $\varkappa \in X\;such\;that\;\varkappa_{n}\to \varkappa$ as *n* → ∞. Now, we investigate that ϰ is the fixed point of *F*. By applying Eqs (28) and (29) and conditions (IFP4),(IFP8), we have

$$\begin{array}{c} M\left(\varkappa ,F\varkappa ,\alpha \right)\geq M\left(\varkappa ,\varkappa_{n},\frac{\alpha }{5Q\left(\varkappa ,\varkappa_{n}\right)}\right)*M\left(F\varkappa_{n-1},F\varkappa_{n},\frac{\alpha }{5W\left(\varkappa_{n},\varkappa_{n+1}\right)}\right)\\ {}*M\left(F\varkappa_{n},F\varkappa_{n+1},\frac{\alpha }{5E\left(\varkappa_{n+1},\varkappa_{n+2}\right)}\right)*M\left(F\varkappa_{n+1},F\varkappa_{n+2},\frac{\alpha }{5R\left(\varkappa_{n+2},\varkappa_{n+3}\right)}\right)*M\left(F\varkappa_{n+2},F\varkappa ,\frac{\alpha }{5T\left(\varkappa_{n+3},F\varkappa \right)}\right)\\ \geq M\left(\varkappa ,\varkappa_{n},\frac{\alpha }{5Q\left(\varkappa ,\varkappa_{n}\right)}\right)*M\left(\varkappa_{n-1},\varkappa_{n},\frac{\alpha }{5pW\left(\varkappa_{n},\varkappa_{n+1}\right)}\right)\\ {}*M\left(\varkappa_{n},\varkappa_{n+1},\frac{\alpha }{5pE\left(\varkappa_{n+1},\varkappa_{n+2}\right)}\right)*M\left(\varkappa_{n+1},\varkappa_{n+2},\frac{\alpha }{5pR\left(\varkappa_{n+2},\varkappa_{n+3}\right)}\right)*M\left(\varkappa_{n+2},\varkappa ,\frac{\alpha }{5pT\left(\varkappa_{n+3},F\varkappa \right)}\right), \end{array}$$

and

$$N\left(\varkappa ,F\varkappa ,\alpha \right)\leq N\left(\varkappa ,\varkappa_{n},\frac{\alpha }{5Q\left(\varkappa ,\varkappa_{n}\right)}\right)\Delta \;N\left(F\varkappa_{n-1},F\varkappa_{n},\frac{\alpha }{5W\left(\varkappa_{n},\varkappa_{n+1}\right)}\right)\Delta \;N\left(F\varkappa_{n},F\varkappa_{n+1},\frac{\alpha }{5E\left(\varkappa_{n+1},\varkappa_{n+2}\right)}\right)$$

$$\Delta \;N\left(F\varkappa_{n+1},F\varkappa_{n+2},\frac{\alpha }{5R\left(\varkappa_{n+2},\varkappa_{n+3}\right)}\right)\Delta \;N\left(F\varkappa_{n+2},F\varkappa ,\frac{\alpha }{5T\left(\varkappa_{n+3},F\varkappa \right)}\right)$$

$$\leq N\left(\varkappa ,\varkappa_{n},\frac{\alpha }{5Q\left(\varkappa ,\varkappa_{n}\right)}\right)\Delta \;N\left(\varkappa_{n-1},\varkappa_{n},\frac{\alpha }{5pW\left(\varkappa_{n},\varkappa_{n+1}\right)}\right)\Delta \;N\left(\varkappa_{n},\varkappa_{n+1},\frac{\alpha }{5pE\left(\varkappa_{n+1},\varkappa_{n+2}\right)}\right)$$

$$\Delta \;N\left(\varkappa_{n+1},\varkappa_{n+2},\frac{\alpha }{5pR\left(\varkappa_{n+2},\varkappa_{n+3}\right)}\right)\Delta \;N\left(\varkappa_{n+2},\varkappa ,\frac{\alpha }{5pT\left(\varkappa_{n+3},F\varkappa \right)}\right).$$

Letting *n* → ∞ in the above inequalities, we deduce Fϰ=ϰ,i.e.,ϰ is a fixed point of *F*. By applying the inequalities (2) and (3), it is easy to show that ϰ is a unique fixed point of *F*.

**Corollary 3.1** Let (*X*, *M*, *N*, *, Δ) be a complete IFCHMS and *Q*: *X* × *X* → [1, ∞) such that

$$\underset{\alpha \to \infty }{\text{lim}}\;M\left(\varkappa ,d,\alpha \right)=1,\;and\underset{\alpha \to \infty }{\text{lim}}\;N\left(\varkappa ,d,\alpha \right)=0,\;for\;all\;\alpha >0\;and\;\varkappa ,d\in X.$$

Let *F*: *X* → *X* be a mapping satisfying

$$M\left(F\varkappa ,Fd,q\alpha \right)\geq M\left(\varkappa ,d,\alpha \right),\;\text{for}\;\text{all}\;\alpha >0\;\text{and}\;\varkappa ,d\in X,$$ (30)

$$N\left(F\varkappa ,Fd,q\alpha \right)\leq N\left(\varkappa ,d,\alpha \right),\;\text{for}\;\text{all}\;\alpha >0\;\text{and}\;\varkappa ,d\in X,$$ (31)

where 0 < *p <* 1 Furthermore, if, for $\varkappa_{0}\in X$ and *n*, *q* ∈ {1,2,3,⋯}, It holds $b\left(\varkappa_{n},\varkappa_{n+q}\right)<\frac{1}{p}$ where $\varkappa_{n}=F^{n}\varkappa_{0},$ then *F* has a unique fixed point.

**Proof:** It is immediate if we take $W\left(e,\kappa \right)=Q\left(e,\kappa \right),\;E\left(\kappa ,g\right)=Q\left(\kappa ,g\right),\;R\left(g,\varpi \right)=Q\left(g,\varpi \right),T\left(\varpi ,d\right)=Q\left(\varpi ,d\right)$ in Theorem 3.1.

**Corollary 3.2** Let (*X*, *M*, *N*, *, Δ) be a complete IFEHbMS and *Q*: *X × X →* [1, ∞) such that

$$\underset{\alpha \to \infty }{\text{lim}}\;M\left(\varkappa ,d,\alpha \right)=1,\;and\underset{\alpha \to \infty }{\text{lim}}\;N\left(\varkappa ,d,\alpha \right)=0,\;for\;all\;\alpha >0\;and\;\varkappa ,d\in X.$$

Let *F*: *X* → *X* be a mapping satisfying

$$M\left(F\varkappa ,Fd,q\alpha \right)\geq M\left(\varkappa ,d,\alpha \right),\;for\;all\;\alpha >0\;and\;\varkappa ,d\in X,$$

$$N\left(F\varkappa ,Fd,q\alpha \right)\leq N\left(\varkappa ,d,\alpha \right),\;for\;all\;\alpha >0\;and\;\varkappa ,d\in X,$$ (32)

where 0 < *p <* 1. Furthermore, if, for $\varkappa_{0}\in X$ and *n*, *q*, ∈ {1,2,3,⋯}, It holds $b\left(\varkappa_{n},\varkappa_{n+q}\right)<\frac{1}{p}$ where $\varkappa_{n}=F^{n}\varkappa_{0},$ then *F* has a unique fixed point.

**Proof:** It is immediate if we take $Q\left(\varkappa ,e\right)=W\left(e,\kappa \right)=E\left(\kappa ,g\right)=R\left(g,\varpi \right)=T\left(\varpi ,d\right)=b\left(\varkappa ,d\right)$ in Theorem 3.1.

**Corollary 3.3:** Let $Q,W,R:X\times X\to \left[1,\frac{1}{k}\right)$ are three non comparable functions where *k* ∈ (0,1) and (*X*, *M*, *N*, *, Δ) is a complete IFTCMS, such that

$$\underset{\alpha \to \infty }{\text{lim}}\;M\left(\varkappa ,d,\alpha \right)=1,\;and\underset{\alpha \to \infty }{\text{lim}}\;N\left(\varkappa ,d,\alpha \right)=0,\;for\;all\;\alpha >0\;and\;\varkappa ,d\in X.$$

Let *F*: *X* → *X* be a mapping satisfying

$$M\left(F\varkappa ,Fd,q\alpha \right)\geq M\left(\varkappa ,d,\alpha \right),\;for\;all\;\alpha >0\;and\;\varkappa ,d\in X,$$

$$N\left(F\varkappa ,Fd,q\alpha \right)\leq N\left(\varkappa ,d,\alpha \right),\;for\;all\;\alpha >0\;and\;\varkappa ,d\in X,$$

then *F* has a unique fixed point.

**Proof**: It is immediate if we take *g = ϖ = d* and *γ + δ+ w = r′* in Theorem 3.1.

**Example 3.4** Let *X =* [0,1]. Define *M*: *X* × *X* × [0, ∞) → [0,1] as

$$M\left(\varkappa ,d,\alpha \right)=e^{-\frac{{\left|\varkappa -d\right|}^{\varpi }}{\alpha }},\;for\;all\;\alpha >0\;and\;\varpi \geq 1,$$

$$N\left(\varkappa ,d,\alpha \right)=1-e^{-\frac{{\left|\varkappa -d\right|}^{\varpi }}{\alpha }},\;for\;all\;\alpha >0\;and\;\varpi \geq 1,$$

with the CTN * such that *α*_1_ * *α*_2_ = *α*_1_*α*_2_, and CTCN Δ such that *α*_1_ Δ *α*_2_ = max{*α*_1_, *α*_2_}, Then (*X*, *M*, *N*, *, Δ) is a complete IFPCMS with non-comparable control functions $Q\left(\varkappa ,d\right)=1+\varkappa +d,\;W\left(\varkappa ,d\right)=1+\varkappa^{2}+d^{2},\;E\left(\varkappa ,d\right)=1+\frac{\varkappa }{d},\;R\left(\varkappa ,d\right)=1+\frac{d}{\varkappa }\;and\;T\left(\varkappa ,d\right)=1+\varkappa^{2}+d$.

Define *F*: *X* → *X* by $F\varkappa =\frac{\varkappa }{6}.$ Consider

$$M\left(F\varkappa ,Fd,q\alpha \right)=M\left(\frac{\varkappa }{6},\frac{d}{6},q\alpha \right)=e^{-\frac{{\left|\frac{\varkappa }{6}-\frac{d}{6}\right|}^{\varpi }}{q\alpha }}=e^{-\frac{{\left|\varkappa -d\right|}^{\varpi }}{6^{\varpi }q\alpha }}\geq e^{-\frac{{\left|\varkappa -d\right|}^{\varpi }}{\alpha }}=M\left(\varkappa ,d,\alpha \right).$$

**Fig 1:**
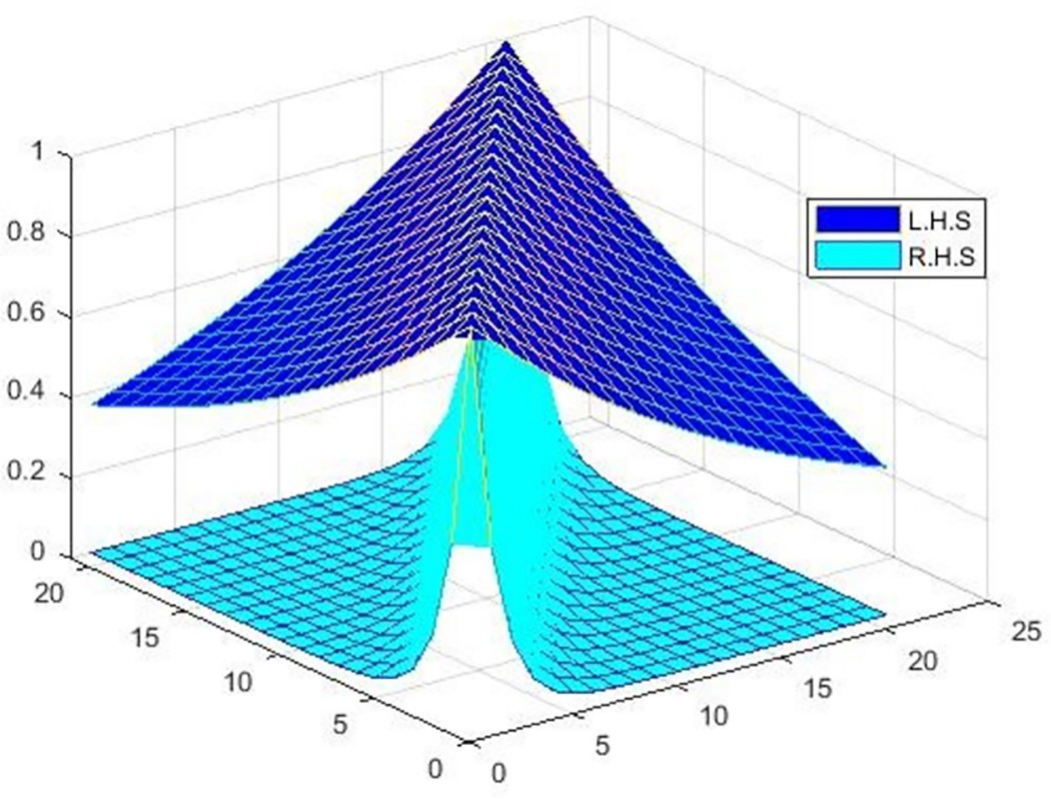
Shows the graphical behavior of an inequality $M\left(F\varkappa ,Fd,q\alpha \right)\geq M\left(\varkappa ,d,\alpha \right)$ for *ϖ* = 1, *α* = 1, and *q* = 0.01.

Now, we show that $M\left(F\varkappa ,Fd,q\alpha \right)\geq M\left(\varkappa ,d,\alpha \right)$ by plotting the below Fig 1.

$$N\left(F\varkappa ,Fd,q\alpha \right)=N\left(\frac{\varkappa }{6},\frac{d}{6},q\alpha \right)=1-e^{-\frac{{\left|\frac{\varkappa }{6}-\frac{d}{6}\right|}^{\varpi }}{q\alpha }}=1-e^{-\frac{{\left|\varkappa -d\right|}^{\varpi }}{6^{\varpi }q\alpha }}\leq 1-e^{-\frac{{\left|\varkappa -d\right|}^{\varpi }}{\alpha }}=M\left(\varkappa ,d,\alpha \right).$$

**Fig 2:**
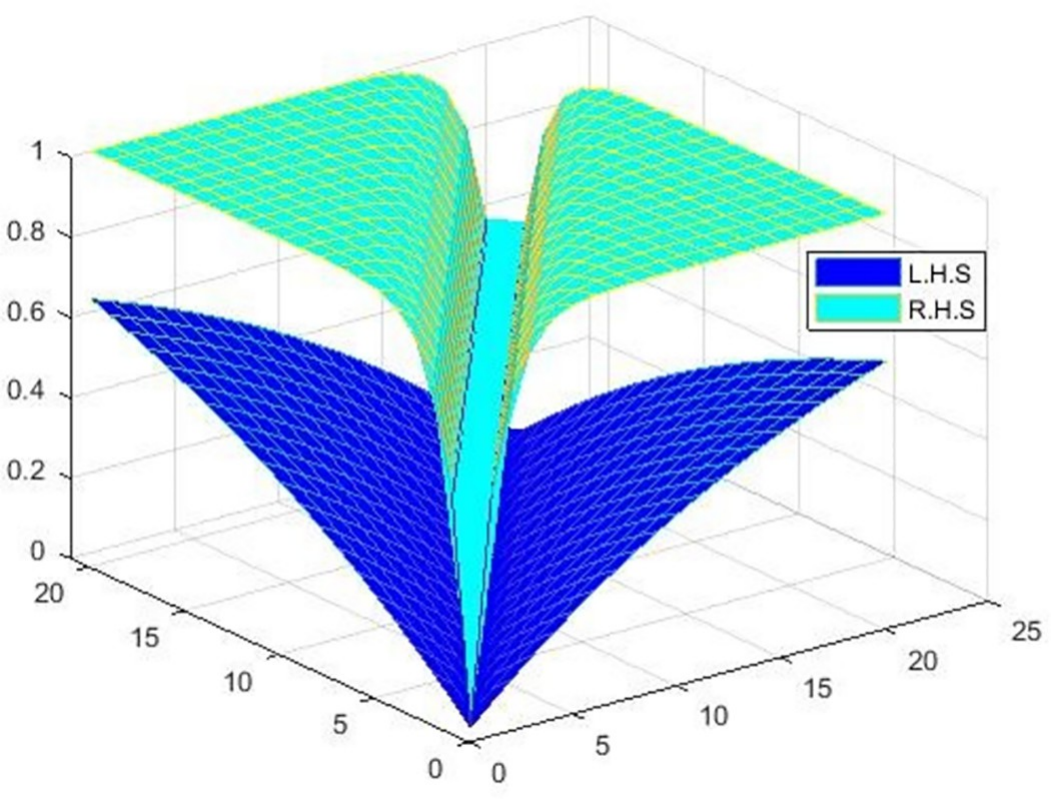
Shows the graphical behavior of an inequality $N\left(F\varkappa ,Fd,q\alpha \right)\leq N\left(\varkappa ,d,\alpha \right)$ for *ϖ* = 1, *α* = 1, and *q* = 0.01.

Now, we show that $N\left(F\varkappa ,Fd,q\alpha \right)\leq N\left(\varkappa ,d,\alpha \right)$ by plotting the below Fig 2.

**Fig 3:**
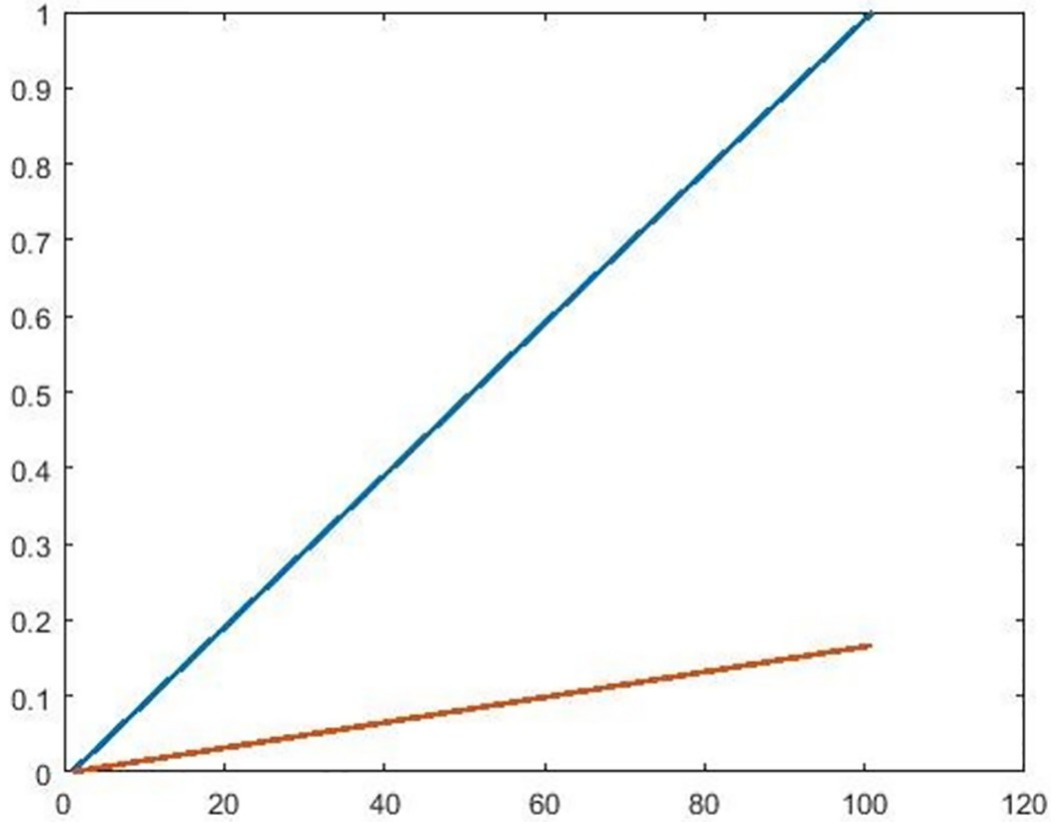
Shows that 0 is a unique fixed point of *F*.

Hence, by Theorem 3.1, *F* has unique fixed point, which is 0 as shown in the below Fig 3.

## 4. Application to dynamic market equilibrium

In this section, we demonstrate how our previously proven result can be used to identify the unique solution to an integral equation in dynamic market equilibrium the field of Economics. Supply *Q*_*β*_ and demand *Q*_*d*_, in many markets, current prices and pricing trends (whether prices are rising or dropping and whether they are rising or falling at an increasing or decreasing rate) have an impact. The economist, therefore, wants to know what the current price is *P*(*α*), the first derivative $\frac{dP(\alpha )}{d\alpha \;}$, and the second derivative $\frac{d^{2}P(\alpha )}{{d\alpha }^{2}}$. Assume

$$Q_{\beta \;}=g_{1}+\;\gamma_{1}P\left(\alpha \right)+e_{1}\frac{dP(\alpha )}{d\alpha }+\;\varkappa_{1}\frac{d^{2}P(\alpha )}{{d\alpha }^{2}},$$

$$Q_{d\;}=g_{2}+\;\gamma_{2}P\left(\alpha \right)+e_{2}\frac{dP(\alpha )}{d\alpha }+\;\varkappa_{2}\frac{d^{2}P(\alpha )}{{d\alpha }^{2}},$$

where *g*_1_, *g*_2_, *γ*_1_, *γ*_2_, *e*_1_ and *e*_2_ are constants. If pricing clears the market at each point in time, we can conclude that the market is dynamically stable. In equilibrium, *Q*_*β*_ *= Q*_*d*._ So

$$g_{1}+\;\gamma_{1}P\left(\alpha \right)+e_{1}\frac{dP(\alpha )}{d\alpha }+\;\varkappa_{1}\frac{d^{2}P(\alpha )}{{d\alpha }^{2}}=g_{2}+\;\gamma_{2}P\left(\alpha \right)+e_{2}\frac{dP(\alpha )}{d\alpha }+\;\varkappa_{2}\frac{d^{2}P(\alpha )}{{d\alpha }^{2}}.$$

Since

$$\left(\varkappa_{1}-\;\varkappa_{2\;}\right)\frac{d^{2}P(\alpha )}{{d\alpha }^{2}}+\left(e_{1}-\;e_{2}\right)d\frac{dP\left(\alpha \right)}{d\alpha }+\left(\gamma_{1}-\;\gamma_{2}\right)P\left(\alpha \right)=-\left(g_{1}-\;g_{2}\right).$$

Letting $\varkappa =\varkappa_{1}-\;\varkappa_{2\;},\;e=e_{1}-\;e_{2\;},\;\gamma =\gamma_{1}-\;\gamma_{2\;}and\;g=g_{1}-\;g_{2}$ in above, we have

$$\varkappa \frac{d^{2}P(\alpha )}{{d\alpha }^{2}}+e\frac{dP(\alpha )}{d\alpha }+\gamma P\left(\alpha \right)=-g.$$

Dividing through by $\varkappa ,\;P\;\left(\alpha \right)$ is governed by the following initial value problem

P''+eϰP''+γϰPα=-gϰP0=0P'0=0, (33)

where $\frac{e^{2}}{\varkappa }=\frac{4\gamma \;}{\varkappa \;}\;and\;\frac{\gamma }{e\;}=\mu \;$ is a continuous function. It is straightforward to demonstrate that the problem (34) is identical to the integral equation:

$$P\left(\alpha \right)=\int_{0}^{T}\psi \left(\alpha ,\;r\right)F(\alpha ,r,P(r))\;dr,$$

where *ψ*(*α*, *r*) is Green‘s function given by

$$\psi \left(\alpha ,r\right)=\left\{\begin{array}{c} re^{\frac{\mu }{2}\left(\alpha -r\right)}\;\text{if}\;0\;\leq r\;\leq \alpha \leq T\\ \alpha e^{\frac{\mu }{2}(r-\alpha )}\;\text{if}\;0\leq \alpha \leq r\leq \alpha \leq T. \end{array}\right.$$

The integral equation has a solution, as we shall demonstrate:

$$P\left(\alpha \right)=\;\int_{0}^{T}G(\alpha ,r,\;P\left(r\right))dr.$$ (34)

Let *X = C* ([0, *T*]) set of real continuous functions defined on [0, *T*] for *α >* 0, we define

Mh,ϰ,α=0,ifα=0supα∈[0,T]minh,ϰ+αmaxh,ϰ+α,otherwise.

Nh,ϰ,α=1,ifα=01-supα∈[0,T]minh,ϰ+αmaxh,ϰ+α,otherwise.

For all h,ϰϵX with *α*_1_ * *α*_2_ = *α*_1_*α*_2_, and *α*_1_ Δ *α*_2_ = max{*α*_1_, *α*_2_}. Define *Q*, *W*, *E*, *R*, *T*: *X* × *X* → [1, ∞). As

$$Q\left(\varkappa ,d\right)=1+\varkappa +d,$$

$$W\left(\varkappa ,d\right)=1+\varkappa^{2}+d^{2},\;E\left(\varkappa ,d\right)=1+\frac{\varkappa }{d},\;R\left(\varkappa ,d\right)=1+\frac{d}{\varkappa }\;and\;T\left(\varkappa ,d\right)=1+\varkappa^{2}+d.$$

It is easy to prove that (*X*, *M*, *N*, *, Δ) is a complete IFPCMS and *F*: *X* → *X* defined by

$$FP\left(\alpha \right)=\int_{0}^{T}G\;(\alpha ,r,P\left(r\right)dr.$$

**Theorem 4.1** Assume an Eq (35) and let that

**(i)** *G*: [0, *T*] × [0, *T*] → ℝ^+^ is continuous function;
**(ii)** there exists a continuous function $\psi :\left[0,\;T\right]\times [0,\;T]\to R^{+}$ such that ${sup}_{\alpha \in [0,T]}\int_{0}^{T}\psi \left(\alpha ,r\right)dr\;\geq 1;$
**(iii)** $\text{max}\;\left\{G\left(\alpha ,r,h\left(r\right)\right)-G\left(\alpha ,r,\varkappa \left(r\right)\right)\right\}\geq \psi \left(\alpha ,r\right)\text{max}\;\left\{h\left(r\right),\;\varkappa \left(r\right)\right\}\;and\;\text{min}\;\left\{G\left(\alpha ,r,h\left(r\right)\right)-G\left(\alpha ,r,\varkappa \left(r\right)\right)\right\}\;\geq \psi \left(\alpha ,r\right)\text{min}\;\left\{h\left(r\right),\;\varkappa \left(r\right)\right\}.$

Then, the integral Eq (35) has a unique solution.

**Proof:** For h,ϰ∈X, by using of assumptions (i) to (iii), we have

$$M\left(Fh,F\varkappa ,\alpha \right)={sup}_{\alpha \epsilon \left[0,T\right]}\frac{\text{min}\;\left\{\int_{0}^{T}G(\alpha ,r,h(r))\;dr,\int_{0}^{T}G(\alpha ,r,\varkappa (r))\;dr\;\right\}+\alpha }{\text{max}\;\left\{\int_{0}^{T}G(\alpha ,r,h(r))\;dr,\int_{0}^{T}G(\alpha ,r,\varkappa (r))\;dr\right\}+\alpha }$$

$$=\;{sup}_{\alpha \epsilon \left[0,T\right]}\frac{\int_{0}^{T}\text{min}\;\left\{G\left(\alpha ,r,h\left(r\right)\right),G\left(\alpha ,r,\varkappa \left(r\right)\right)\right\}\mathrm{dr}\;+\alpha }{\int_{0}^{T}\text{max}\;\left\{G\left(\alpha ,r,h\left(r\right)\right),G\left(\alpha ,r,\varkappa \left(r\right)\right)\right\}\mathrm{dr}\;+\alpha }$$

$$\geq {sup}_{\alpha \epsilon \left[0,T\right]}\frac{\int_{0}^{T}\psi \left(\alpha ,r\right)\text{min}\;\left\{h\left(r\right),\varkappa \left(r\right)\right\}\mathrm{dr}\;+\alpha }{\int_{0}^{T}\psi \left(\alpha ,r\right)\text{max}\;\left\{h\left(r\right),\varkappa \left(r\right)\right\}\mathrm{dr}\;+\alpha }$$

$$\geq {sup}_{\alpha \epsilon \left[0,T\right]}\frac{\text{min}\;\left\{h\left(r\right),\varkappa \left(r\right)\right\}\int_{0}^{T}\psi \left(\alpha ,r\right)\mathrm{dr}\;+\alpha }{\text{max}\;\left\{h\left(r\right),\varkappa \left(r\right)\right\}\int_{0}^{T}\psi \left(\alpha ,r\right)\mathrm{dr}\;+\alpha }$$

$$\geq \left(\frac{\text{min}\;\left\{h\left(r\right),\;\varkappa \left(r\right)\right\}+\alpha }{\text{max}\;\left\{h\left(r\right),\;\varkappa \left(r\right)\right\}+\alpha }\right)=M\left(h,\varkappa ,\alpha \right),$$

and

$$N\left(Fh,F\varkappa ,\alpha \right)=1-{sup}_{\alpha \epsilon \left[0,T\right]}\frac{\text{min}\;\left\{\int_{0}^{T}G(\alpha ,r,h(r))\;dr,\int_{0}^{T}G(\alpha ,r,\varkappa (r))\;dr\;\right\}+\alpha }{\text{max}\;\left\{\int_{0}^{T}G(\alpha ,r,h(r))\;dr,\int_{0}^{T}G(\alpha ,r,\varkappa (r))\;dr\right\}+\alpha }$$

$$=\;1-{sup}_{\alpha \epsilon \left[0,T\right]}\frac{\int_{0}^{T}\text{min}\;\left\{G\left(\alpha ,r,h\left(r\right)\right),G\left(\alpha ,r,\varkappa \left(r\right)\right)\right\}\mathrm{dr}\;+\alpha }{\int_{0}^{T}\text{max}\;\left\{G\left(\alpha ,r,h\left(r\right)\right),G\left(\alpha ,r,\varkappa \left(r\right)\right)\right\}\mathrm{dr}\;+\alpha }$$

$$\leq 1-{sup}_{\alpha \epsilon \left[0,T\right]}\frac{\int_{0}^{T}\psi \left(\alpha ,r\right)\text{min}\;\left\{h\left(r\right),\varkappa \left(r\right)\right\}\mathrm{dr}\;+\alpha }{\int_{0}^{T}\psi \left(\alpha ,r\right)\text{max}\;\left\{h\left(r\right),\varkappa \left(r\right)\right\}\mathrm{dr}\;+\alpha }$$

$$\leq 1-{sup}_{\alpha \epsilon \left[0,T\right]}\frac{\text{min}\;\left\{h\left(r\right),\varkappa \left(r\right)\right\}\int_{0}^{T}\psi \left(\alpha ,r\right)\mathrm{dr}\;+\alpha }{\text{max}\;\left\{h\left(r\right),\varkappa \left(r\right)\right\}\int_{0}^{T}\psi \left(\alpha ,r\right)\mathrm{dr}\;+\alpha }$$

$$\leq 1-\left(\frac{\text{min}\;\left\{h\left(r\right),\;\varkappa \left(r\right)\right\}+\alpha }{\text{max}\;\left\{h\left(r\right),\;\varkappa \left(r\right)\right\}+\alpha }\right)=N\left(h,\varkappa ,\alpha \right).$$

Thus $M\left(Fh,\;F\varkappa ,\;q\alpha \right)\geq M\left(h,\varkappa ,\;\alpha \right)\;and\;N\left(Fh,\;F\varkappa ,\;q\alpha \right)\leq N\left(h,\varkappa ,\;\alpha \right)\;for\;all\;h,\varkappa \in X,$ and all conditions of Theorem 3.1 are satisfied. Therefore, Eq (34) has a unique fixed point.

## 5. Application to a satellite web coupling problem

We use Theorem 3.1 to solve a satellite web coupling boundary value problem [20] since fixed point techniques have been applied to a variety of real-world challenges. A thin sheet linking two cylinder-shaped satellites is an ideal representation of a satellite web coupling. The following non-linear boundary value problem is caused by the radiation from the web coupling issue between two satellites:

$$-\frac{d^{2}w}{dt^{2}}=\mu w^{4},\;0<t<1,\;w\left(0\right)=w\left(1\right)=0,$$ (35)

where *w*(*t*) shows the temperature of radiation at any point $t\;\in \;\left[0,1\right],\;\mu =\frac{2al^{2}K^{3}}{\psi h}>0$ is a non-dimensional positive constant, *K* is the constant absolute temperature of both satellites, while heat is radiated from the surface of the web into space at 0 absolute temperature, *l* is the distance between two satellites, *a* is a positive constant describing the radiation properties of the surface of the web, factor 2 is required because there is radiation from both the top and bottom surfaces, *ψ* is thermal conductivity, and *h* is the thickness. The Green function

gt,ψ=t1-ψ,0<t<ψψ1-t,ψ<t<1.

Eq (35) is equivalent to

$$w\left(t\right)=1-\mu \int_{0}^{1}g\left(t,\psi \right)w^{4}\left(\psi \right)d\psi .$$

Let *X* = ℛ[0,1] be a set of Riemann integrable functions defined on [0, 1]. we define

$$M\left(h,\varkappa ,t\right)={sup}_{t\in [0,1]}e^{-\;\frac{{\left|h-\varkappa \right|}^{p}}{t}},$$

$$N\left(h,\varkappa ,t\right)=1-{sup}_{t\in \left[0,1\right]}e^{-\;\frac{{\left|h-\varkappa \right|}^{p}}{t}},$$

for all h,ϰ∈X with the CTN ′*′ such that *α*_1_ * *α*_2_ = *α*_1_*α*_2_, and Δ is a CTCN such that *α*_1_ Δ *α*_2_ = max{*α*_1_, *α*_2_}. Define *Q*, *W*, *E*, *R*, *T*: *X × X* → [1, ∞) by

$$Q\left(\varkappa ,d\right)=1+\varkappa +d,\;W\left(\varkappa ,d\right)=1+\varkappa^{2}+d^{2},\;E\left(\varkappa ,d\right)=1+\frac{\varkappa }{d},\;R\left(\varkappa ,d\right)=1+\frac{d}{\varkappa }\;and\;T\left(\varkappa ,d\right)=1+\varkappa^{2}+d.$$

It is easy to prove that *(X*, *M*, *N*, *, Δ) is a complete IFPCMS.

**Theorem 5.1:** Let *f*: *X* → *X* be a self-mapping in a complete IFPCMS, satisfying

$$\left|\varkappa \left(t\right)-e\left(t\right)\right|>0\;\Rightarrow \left|\left(\varkappa^{2}\left(t\right)+e^{2}\left(t\right)\right)\left(\varkappa \left(t\right)+e\left(t\right)\right)\right|\leq \frac{q}{\mu },\;k\in \left(0,8\right).$$ (36)

Then, the satellite web coupling boundary value problem (36) has a unique solution.

**Proof:** Define a self-mapping *f*: *X* → *X* by

$$f\left(\varkappa \left(t\right)\right)=1-\mu \int_{0}^{1}g\left(t,\psi \right)\varkappa^{4}\left(\psi \right)d\psi ,\;\psi \in \left[0,1\right].$$ (37)

Clearly, a solution to the satellite web coupling problem (35) is a fixed point of a self-mapping *f*. However, $\left|\varkappa \left(t\right)-e\left(t\right)\right|>0,$ so

$$M\left(f\left(\varkappa \left(t\right)\right),\;f\left(e\left(t\right)\right),\;qt\right)={sup}_{t\in [0,1]}e^{-\;\frac{{\left|f\left(\varkappa \left(t\right)\right)-f\left(e\left(t\right)\right)\right|}^{p}}{qt}}$$

$$=\;{sup}_{t\in \left[0,1\right]}e^{-\;\frac{{\left|1-\mu \int_{0}^{1}g\left(t,\psi \right)\varkappa^{4}\left(\psi \right)\;d\psi -1+\mu \int_{0}^{1}g\left(t,\psi \right)e^{4}\left(\psi \right)\;d\psi \right|}^{p}}{qt}}$$

$$=\;{sup}_{t\in \left[0,1\right]}e^{-\;\frac{{\left|\mu \left[\int_{0}^{1}g\left(t,\psi \right)\varkappa^{4}\left(\psi \right)\;d\psi -\int_{0}^{1}g\left(t,\psi \right)e^{4}\left(\psi \right)\;d\psi \right]\right|}^{p}}{qt}}$$

$$=\;{sup}_{t\in \left[0,1\right]}e^{-\;\frac{{\left|\mu \left[\int_{0}^{1}\left(\varkappa^{4}\left(\psi \right)-e^{4}\left(\psi \right)\right)g\left(t,\psi \right)\;d\psi \right]\right|}^{p}}{qt}}$$

$$=\;{sup}_{t\in \left[0,1\right]}e^{-\;\frac{{\mu \left|\left[\int_{0}^{1}\left(\varkappa^{2}\left(\psi \right)+e^{2}\left(\psi \right)\right)\left(\varkappa \left(\psi \right)+e\left(\psi \right)\right)g\left(t,\psi \right)d\psi \right]\right|}^{p}}{qt}}$$

$$\geq {sup}_{t\in \left[0,1\right]}e^{-\;\frac{{\left|\varkappa \left(t\right)-e\left(t\right)\right|}^{p}\left[\int_{0}^{t}\psi \left(1-t\right)d\psi +\;\int_{t}^{1}t\left(1-\psi \right)d\psi \right]}{t}}$$

$$\geq {sup}_{t\in [0,1]}e^{-\;\frac{{\left|\varkappa \left(t\right)-e\left(t\right)\right|}^{p}}{t}}=M\left(\varkappa ,e,t\right),$$

$$N\left(f\left(\varkappa \left(t\right)\right),\;f\left(e\left(t\right)\right),\;qt\right)=1-{sup}_{t\in [0,1]}e^{-\;\frac{{\left|f\left(\varkappa \left(t\right)\right)-f\left(e\left(t\right)\right)\right|}^{p}}{qt}}$$

$$=\;1-{sup}_{t\in \left[0,1\right]}e^{-\;\frac{{\left|1-\mu \int_{0}^{1}g\left(t,\psi \right)\varkappa^{4}\left(\psi \right)\;d\psi -1+\mu \int_{0}^{1}g\left(t,\psi \right)e^{4}\left(\psi \right)\;d\psi \right|}^{p}}{qt}}$$

$$=\;1-{sup}_{t\in \left[0,1\right]}e^{-\;\frac{{\left|\mu \left[\int_{0}^{1}g\left(t,\psi \right)\varkappa^{4}\left(\psi \right)\;d\psi -\int_{0}^{1}g\left(t,\psi \right)e^{4}\left(\psi \right)\;d\psi \right]\right|}^{p}}{qt}}$$

$$=\;1-{sup}_{t\in \left[0,1\right]}e^{-\;\frac{{\left|\mu \left[\int_{0}^{1}\left(\varkappa^{4}\left(\psi \right)-e^{4}\left(\psi \right)\right)g\left(t,\psi \right)\;d\psi \right]\right|}^{p}}{qt}}$$

$$=\;1-{sup}_{t\in [0,1]}e^{-\;\frac{{\mu \left|\left[\int_{0}^{1}\left(\varkappa^{2}\left(\psi \right)+e^{2}\left(\psi \right)\right)\left(\varkappa \left(\psi \right)+e\left(\psi \right)\right)g\left(t,\psi \right)d\psi \right]\right|}^{p}}{qt}}$$

$$\leq 1-{sup}_{t\in \left[0,1\right]}e^{-\;\frac{{\left|\varkappa \left(t\right)-e\left(t\right)\right|}^{p}\left[\int_{0}^{t}\psi \left(1-t\right)d\psi +\;\int_{t}^{1}t\left(1-\psi \right)d\psi \right]}{t}}$$

$$\leq 1-{sup}_{t\in [0,1]}e^{-\;\frac{{\left|\varkappa \left(t\right)-e\left(t\right)\right|}^{p}}{t}}=N\left(\varkappa ,e,t\right).$$

Therefore, all the conditions of Theorem 3.1 are satisfied. Hence, *f* has a unique fixed point, and a satellite web coupling problem (36) has a unique solution.

## 6. Discussion and conclusions

In this paper, we introduced the notions of IFTCMS, IFHEbMS, IFHCMS, and IFPCMS as a generalization of several notions existing in the previous literature [1, 2, 9, 14, 15, 18], in which we extended the triangular inequality and used membership and non-membership functions. In the definition of an IFPCMS:

- If we take $W\left(e,\kappa \right)=Q\left(e,\kappa \right),\;E\left(\kappa ,g\right)=Q\left(\kappa ,g\right),\;R\left(g,\varpi \right)=Q\left(g,\varpi \right),T\left(\varpi ,d\right)=Q\left(\varpi ,d\right),$ then it will become the definition of IFCHMS.
- If we take $Q\left(\varkappa ,e\right)=W\left(e,\kappa \right)=E\left(\kappa ,g\right)=R\left(g,\varpi \right)=T\left(\varpi ,d\right)=b\left(\varkappa ,d\right),$ then it will become the definition of IFEHbMS.
- If *g* = *ϖ = d* and *γ + δ + w = r′*, then it will become IFTCMS.
- If *κ = g* = *ϖ = d* and *β + γ + δ + w = t′*, then it will become IFDCMS in [18].
- If *κ = g* = *ϖ = d*, *β + γ + δ + w = t′*, and *W*(*e*, *κ*) = *E*(*κ*, *g*) = *b ≥* 1 then it will become IFbMS in [15].
- If *κ = g* = *ϖ = d*, *β + γ + δ + w = t′*, and *W*(*e*, *κ*) = *E*(*κ*, *g*) = 1 then it will become IFMS in [14].
- Every PCFMS is an IFPCMS of the form (*X*, *M*, 1 –*M*, *, Δ), if we take $Z\Delta d=1-\left(\left(1-\varkappa \right)*\left(1-d\right)\right).$

Further, we proved a Banach fixed point theorem in the framework of IFPCMS that is a most generalized notion in IFMSs theory. Furthermore, we provided several examples for introduced notions and graphical representation to show the existence of fixed point of our main result. At the end, we presented applications to dynamic market equilibrium and satellite web coupling problem. This work is extendable in the context of intuitionistic fuzzy pentagonal controlled cone metric spaces, intuitionistic fuzzy pentagonal controlled partial metric spaces, pentagonal neutrosophic metric spaces and many other structures.
